# GIMAP6 is required for T cell maintenance and efficient autophagy in mice

**DOI:** 10.1371/journal.pone.0196504

**Published:** 2018-05-02

**Authors:** John C. Pascall, Louise M. C. Webb, Eeva-Liisa Eskelinen, Silvia Innocentin, Noudjoud Attaf-Bouabdallah, Geoffrey W. Butcher

**Affiliations:** 1 Laboratory of Lymphocyte Signalling and Development, Babraham Institute, Babraham Research Campus, Cambridge, United Kingdom; 2 Department of Biosciences, Faculty of Biological and Environmental Sciences, University of Helsinki, Helsinki, Finland; University of Alabama at Birmingham, UNITED STATES

## Abstract

The **G**TPases of the **im**munity-**a**ssociated **p**roteins (GIMAP) GTPases are a family of proteins expressed strongly in the adaptive immune system. We have previously reported that in human cells one member of this family, GIMAP6, interacts with the ATG8 family member GABARAPL2, and is recruited to autophagosomes upon starvation, suggesting a role for GIMAP6 in the autophagic process. To study this possibility and the function of GIMAP6 in the immune system, we have established a mouse line in which the *Gimap6* gene can be inactivated by Cre-mediated recombination. In mice bred to carry the CD2Cre transgene such that the *Gimap6* gene was deleted within the T and B cell lineages there was a 50–70% reduction in peripheral CD4^+^ and CD8^+^ T cells. Analysis of splenocyte-derived proteins from these mice indicated increased levels of MAP1LC3B, particularly the lipidated LC3-II form, and S405-phosphorylation of SQSTM1. Electron microscopic measurements of *Gimap6*^*-/-*^ CD4^+^ T cells indicated an increased mitochondrial/cytoplasmic volume ratio and increased numbers of autophagosomes. These results are consistent with autophagic disruption in the cells. However, *Gimap6*^*-/-*^ T cells were largely normal in character, could be effectively activated *in vitro* and supported T cell-dependent antibody production. Treatment *in vitro* of CD4^+^ splenocytes from GIMAP6^fl/fl^ERT2Cre mice with 4-hydroxytamoxifen resulted in the disappearance of GIMAP6 within five days. In parallel, increased phosphorylation of SQSTM1 and TBK1 was observed. These results indicate a requirement for GIMAP6 in the maintenance of a normal peripheral adaptive immune system and a significant role for the protein in normal autophagic processes. Moreover, as GIMAP6 is expressed in a cell-selective manner, this indicates the potential existence of a cell-restricted mode of autophagic regulation.

## Introduction

The AIG1 family of GTPases are a group of proteins found sporadically in various eukaryotic phyla [1]. The first member of the family, termed AIG1 (avrRpt2-induced gene 1), was identified in the plant species *Arabidopsis thaliana*, where it is induced in response to *Pseudomonas syringae* infection [2]. In addition to plants, members of the family have also been identified in a restricted number of other groups including protists [3], coral [4] and molluscs [5,6], (but not in e.g. *Saccharomyces cerevisiae*, *Caenorhabditis elegans* or *Drosophila melanogaster*) and throughout vertebrates [7]. Interestingly, in addition to the induction of AIG1 in *Arabidopsis* by infection, in both coral [4] and molluscs [5,6] AIG1 family members are also induced by pathogenic challenge, suggesting that they may have a significant role in conferring resistance to infection. The link to host defence is further conserved in vertebrates, where the AIG1 family of GTPases is represented by the GIMAP family of proteins and expression of these is most prominent in cells of the adaptive immune system [7].

In mammals, the GIMAP family comprises 7–8 members (species-dependent) which are closely linked at a single locus (chromosome 7 in human, 6 in mouse) [8]. The family can be split into two groups, depending on the presence or absence of membrane-anchoring domains. In mouse, GIMAPs 1, 3 and 5 are membrane-anchored, whereas GIMAPs 4, 6, 7, 8 and 9 are soluble proteins (see [9]). In structural terms, GIMAPs have been placed in the non-Ras class of G proteins alongside septins and dynamins with which they share mechanisms of GTPase activation via molecular dimerization (including heterologous interactions within the GIMAP family). A role in molecular scaffold formation on intracellular membranes has been proposed [10].

Historically, GIMAP5 has attracted the most research attention. Non-functional mutations of *Gimap5* in both rats and mice are associated with severe peripheral T cell lymphopenia and increased susceptibility to autoimmune conditions such as type 1 diabetes and inflammatory bowel disease [11–16]. Interestingly, targeted mutations introduced into the genes encoding other GIMAP membrane-anchored proteins present in mice, namely GIMAP1 [17] and GIMAP3 [18], also have an impact on T cell phenotypes, although that associated with GIMAP3 is only seen clearly in the presence of an additional *Gimap5* mutation [18]. In contrast to what has been seen with the membrane-anchored proteins, mice carrying targeted mutations of the genes encoding two of the soluble GIMAPs show no (GIMAP4) [19] or very minor (GIMAP8) [9] changes in the lymphocyte populations, although T cells isolated from both of these mouse strains show altered kinetics of apoptosis *in vitro* [9,19].

We have previously reported work on another soluble member of the family, GIMAP6. We showed that human GIMAP6 interacts strongly with the autophagy-related ATG8 protein GABARAPL2 in transfected cell lines [20], an effect subsequently confirmed in a high-throughput screening assay [21]. Moreover, GIMAP6 was recruited to autophagosomes upon stimulation of autophagy [20], perhaps via its interaction with GABARAPL2. This suggested either that GIMAP6 was selectively degraded by the autophagic pathway or, more interestingly, that it played a functional role in autophagy. However, we were unable to demonstrate any changes associated with altering the expression of GIMAP6 in cell lines. We also made the unexpected observation that the interaction of mouse GIMAP6 with GABARAPL2 was very weak, which we showed was due to the absence from mouse GIMAP6 of a critical domain required for the strong interaction of the human protein with GABARAPL2 [20].

The absence of a strong interaction between mouse GIMAP6 and GABARAPL2 raises the question of whether GIMAP6 plays any functional role in mice. We have now generated a mouse line carrying a conditional knock-out *Gimap6* allele, and report here that GIMAP6 is required for the maintenance of normal levels of peripheral T cells and that this effect is associated with an impairment of autophagy.

## Results

### Mouse GIMAP6 relocates to autophagosomes on induction of autophagy

**Fig 1:**
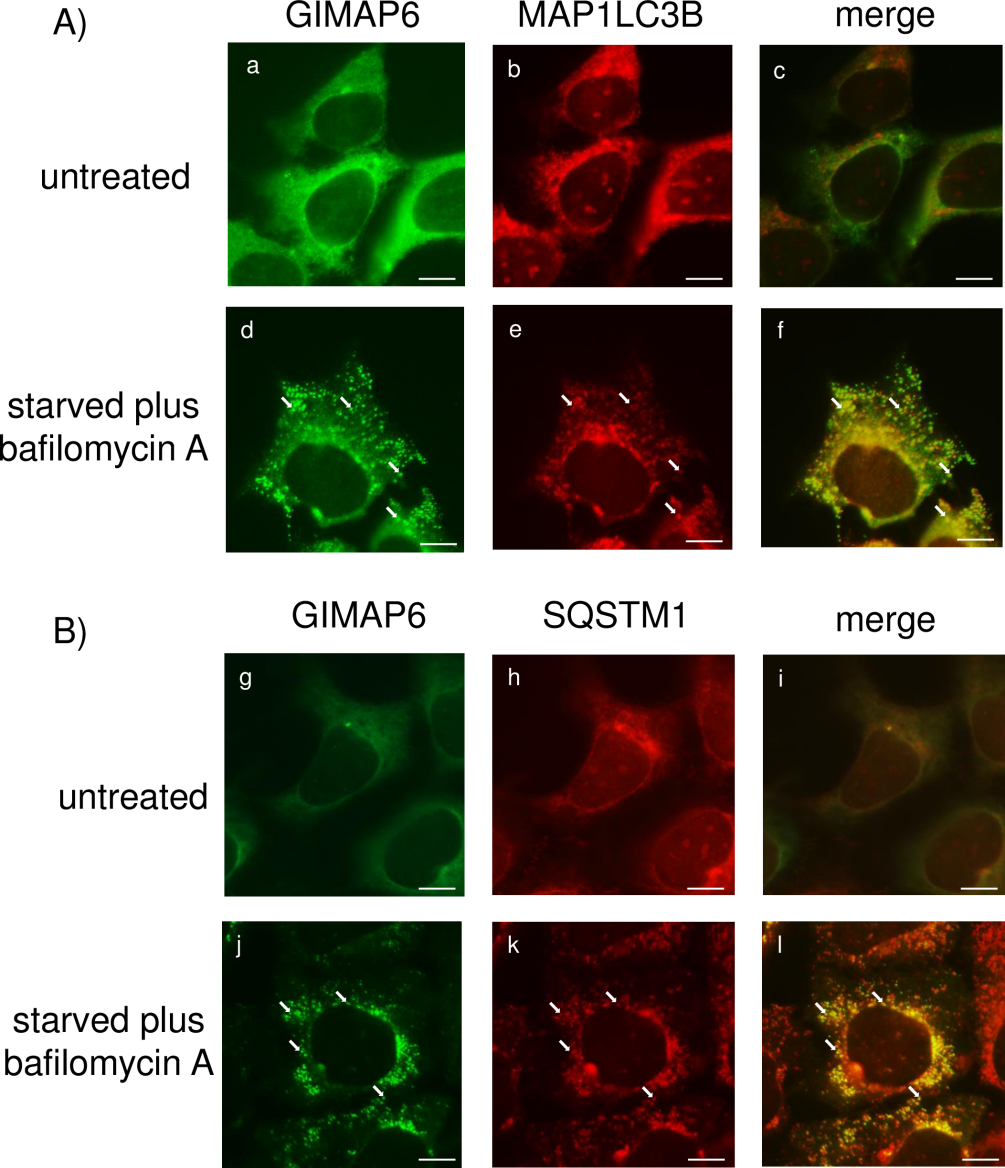
GIMAP6 relocalises to autophagosomes on cell starvation. HEK293 cells stably expressing an N-terminally myc-tagged variant of mouse GIMAP6 were either left untreated or were treated with starvation buffer for 90 minutes. They were then fixed and stained with either (A) a mixture of rat monoclonal antibodies to mouse GIMAP6 and a rabbit polyclonal antibody to MAP1LC3B, or (B) the same mixture of rat monoclonal antibodies to GIMAP6 and a rabbit polyclonal antibody to SQSTM1, each followed by fluorochrome-conjugated secondary antibodies. GIMAP6 is shown false-coloured green and MAP1LC3B or SQSTM1, red. Scale bar represents 10 μm.

As mouse GIMAP6 does not interact strongly with GABARAPL2, the question arises as to whether induction of autophagy results in the relocation of mouse GIMAP6 to vesicular structures, as described previously for the human protein. To address this issue, HEK293 cells were engineered to over-express a myc-tagged variant of mouse GIMAP6. In untreated cells GIMAP6 showed a uniform cytosolic distribution (Fig 1, panels a and g and S1 Fig). Maintenance of cells for 90 minutes either in starvation medium or, to a lesser extent, treatment of the cells with bafilomycin A (BafA), led to the formation of small punctate structures in the cytoplasm (S1 Fig). When the two cell treatments were combined, the GIMAP6 protein showed a strongly staining punctate distribution in the cytoplasm (Fig 1, Panels d and j and S1 Fig). Co-staining of the cells with antibodies to either MAP1LC3B (a classic marker for autophagosomes) (panels b and e) or SQSTM1 (Panels h and k), both of which are recruited to autophagosomes on starvation, demonstrated that they too redistributed from a largely cytosolic to a more punctate appearance upon starvation in the presence of BafA. Moreover, inspection of the spot distribution showed clear (although not complete) co-localisation between GIMAP6 and MAP1LC3B (Panel f), and between GIMAP6 and SQSTM1 (Panel l), indicating that mouse GIMAP6, similar to its human counterpart, relocates, at least in part, to vesicles showing characteristics of autophagosomes. As these results suggested that GIMAP6 might traffic in association with autophagosomal vesicles, the question of whether GIMAP6 could be detected with lysosomes (representing the end of the autophagy flux pathway) was investigated. However, no co-localization of GIMAP6 with the lysosomal marker LAMP1 could be demonstrated either in resting cells or in cells in which autophagy had been stimulated by starvation (S2 Fig).

### Targeted ablation of Gimap6 in lymphocytes

**Fig 2:**
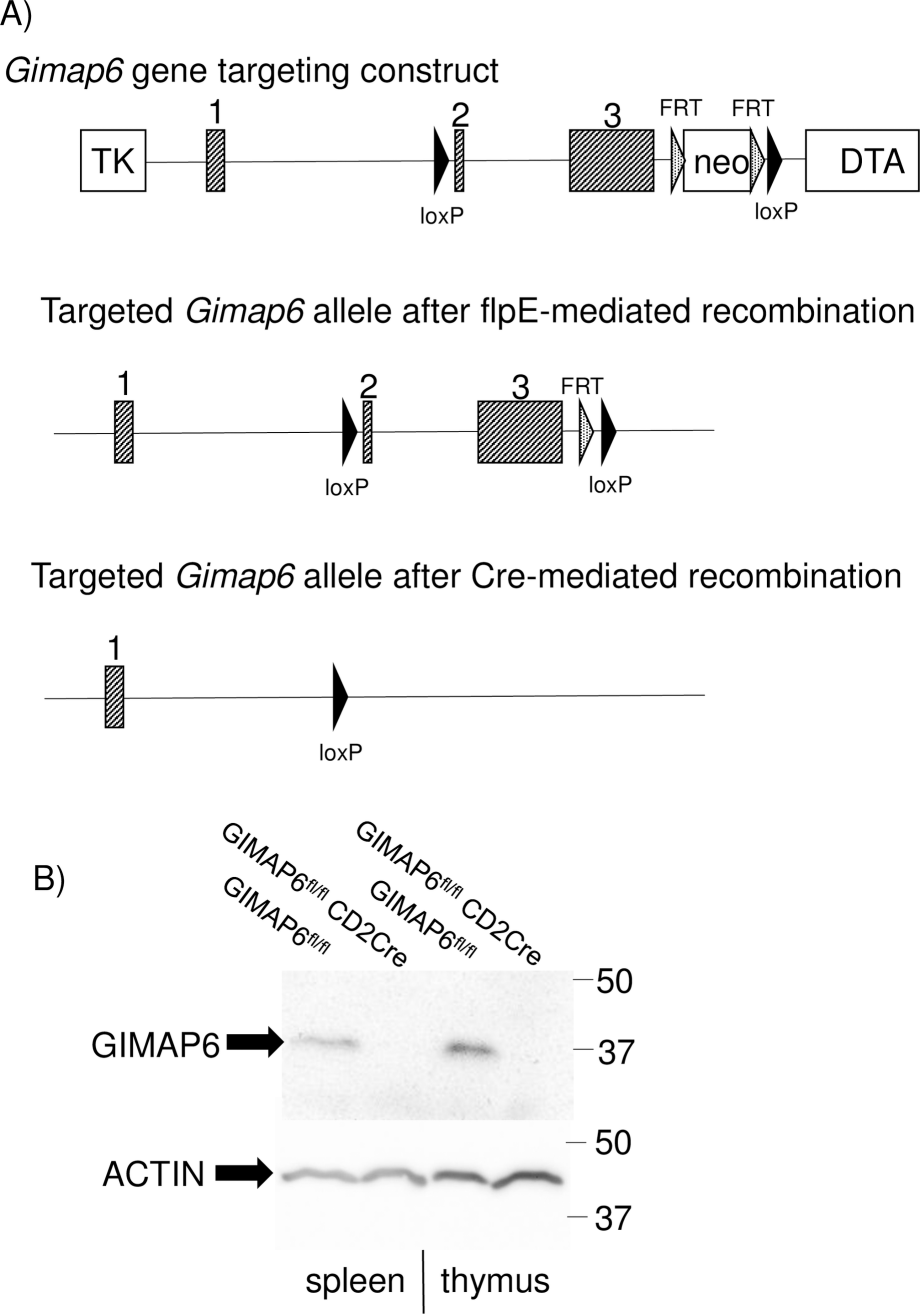
Design and testing of a targeting construct for the mouse Gimap6 gene. Panel A shows the targeting construct in which exons 2 and 3 are flanked by loxP sites, both before and after flpE recombination to remove the selectable neomycin resistance cassette (neo). In addition, DNA fragments encoding thymidine kinase (TK) and diphtheria toxin A chain (DTA) were included in the targeting construct to increase the targeting efficiency in embryonic stem cells. The lower part of panel A shows the integrated DNA fragment after ablation of exons 2 and 3 by Cre-mediated recombination. Panel B shows a Western blot of GIMAP6 (using rat anti-mouse antibody MAC436) and actin on total lymphocytes isolated from thymus or spleen of GIMAP6^fl/fl^ mice, in which the Gimap6 gene is intact, and in GIMAP6^fl/fl^CD2Cre mice, in which Gimap6 should be deleted in the B and T cell populations.

To address the function(s) of GIMAP6 in more detail, a mouse line was generated in which exons two and three of the *Gimap6* gene were flanked by loxP sites (see Fig 2A). On recombination of these sites the altered “gene” would be predicted to encode the first 64 amino acids of GIMAP6 but to lack the remaining 241 residues, including the GTPase domain, and thus would be expected to be non-functional.

GIMAP6, like the rest of the GIMAP family, is prominently expressed in lymphocytes [17]. Therefore, to investigate possible roles of GIMAP6, GIMAP6^fl/fl^ mice were crossed to a *hCD2-iCre* transgene-carrying mouse line. The hCD2 promoter directs Cre recombinase expression to T and B lymphocyte cell lineages [22]. Thus, in the offspring from the cross (termed GIMAP6^fl/fl^CD2Cre animals) it was expected that GIMAP6 ablation would occur in those cell lineages. Thymi and spleens were isolated from the animals and the level of GIMAP6 present in lysates from lymphocytes from these tissues assessed by western blotting (Fig 2B). Whereas in GIMAP6^fl/fl^ animals GIMAP6 polypeptide was detectable as a single band of approximately 37kD in both thymus and spleen, in the tissues from GIMAP6^fl/fl^CD2Cre animals it was undetectable. This indicates that the *Gimap6* allele was being successfully recombined in the GIMAP6^fl/fl^CD2Cre animals, thereby eliminating expression of the protein.

### GIMAP6 ablation results in the accumulation of MAP1LC3B form II and phosphorylated SQSTM1

**Fig 3:**
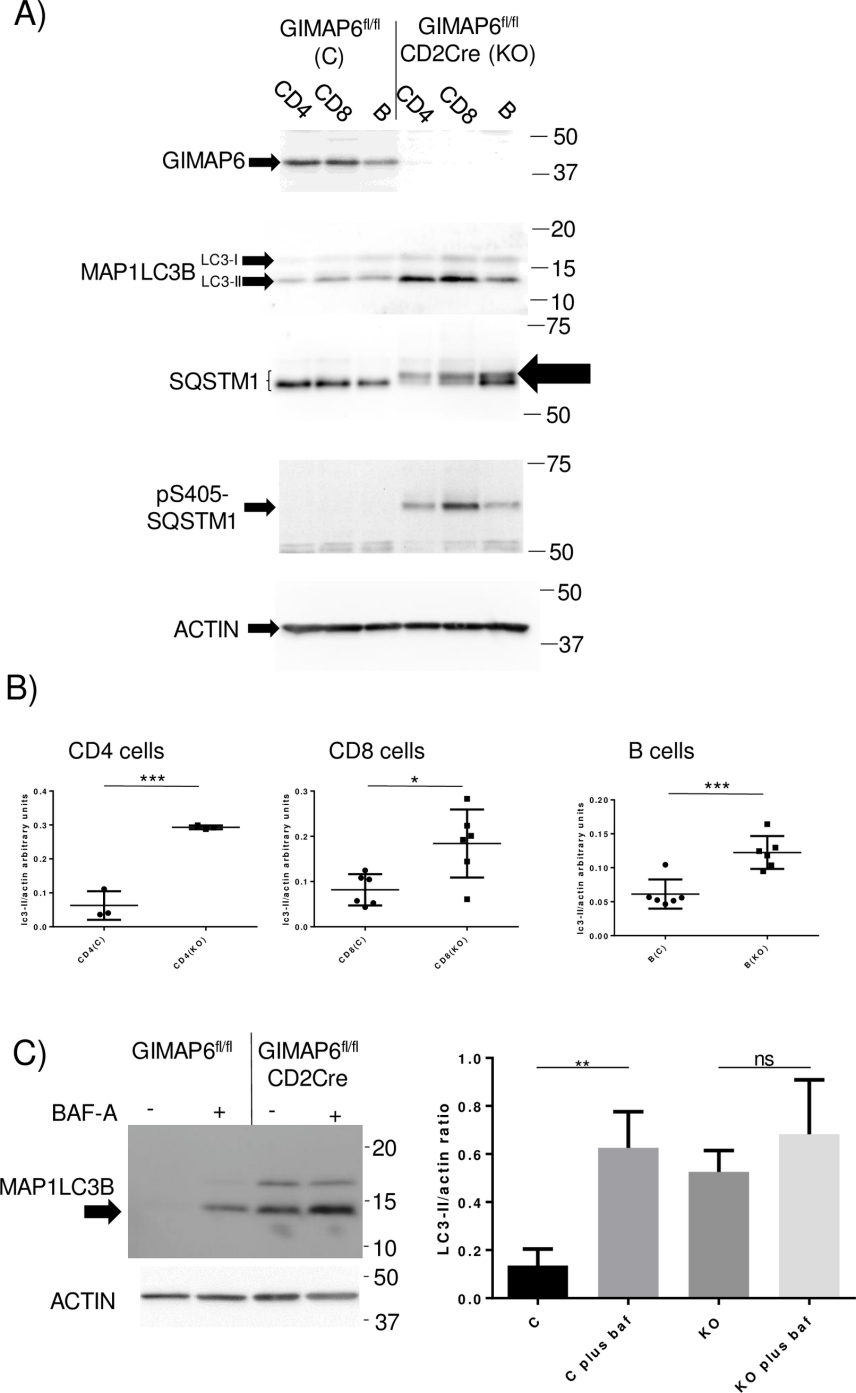
Expression levels of proteins in enriched lymphocyte fractions from GIMAP6^fl/fl^ and GIMAP6^fl/fl^ CD2Cre mice. Panel A) CD4^+^, CD8^+^ and B cell enriched fractions were isolated from splenocytes from GIMAP6^fl/fl^ and GIMAP6^fl/fl^CD2Cre mice. Total cell lysates were prepared and analysed by SDS polyacrylamide gel electrophoresis and western blotting. The antibody sources can be found in the Materials and Methods section. The mobility of co-electrophoresed size markers is indicated. Immunoreactive protein species corresponding to phospho-S405 SQSTM1, a slower migrating SQSTM1 form, and the LC3-I and LC3-II variants of MAP1LC3B are indicated by arrows. All samples were analysed in at least two separate experiments. Panel B) Cellular populations were purified from single mice and analysed as in Panel A. The ratio of LC3-II to actin was determined from gel scans. For CD4^+^ cells n = 3; for CD8^+^ and B cells n = 6. P values are as follows: *p<0.05, ***p<0.001. Panel C) left-hand panel: freshly isolated splenic CD4^+^ cells from GIMAP6^fl/fl^ and GIMAP6^fl/fl^CD2Cre mice (four mice pooled per group) were cultured with or without 100nM bafilomycin A (BafA) for 5 hours before preparation of cell lysates and analysis by Western blotting. The LC3-II species is arrowed (representative of four separate experiments). Panel C) right-hand panel: LC3-II to actin ratios were measured for four separate experiments. Results are presented graphically. C—cells from GIMAP6^fl/fl^ animals; KO—cells from GIMAP6^fl/fl^CD2cre mice. P values are as follows: **p<0.01; ns p>0.05. n = 4.

The immunological effects of ablation of individual GIMAP proteins in knockout models has been well-studied; by contrast, the biochemical pathways through which the proteins function mechanistically remain ill-defined. Although we have previously failed to demonstrate any functional effects of increasing cellular levels of GIMAP6 in established human cell lines, despite demonstrating localisation of the protein to autophagosomes upon starvation of those cells [20], we were interested to determine whether GIMAP6 might have an effect on the process of autophagy in primary cells. CD4^+^ T-, CD8^+^ T- and B cell-enriched populations were isolated from the spleens of GIMAP6^fl/fl^ (control) and GIMAP6^fl/fl^CD2Cre (knockout) mice, cellular lysates prepared and the proteins analysed by western blotting. Immunoblots using monoclonal antibodies to mouse GIMAP6 confirmed the absence of the protein from knockout lymphocyte sub-population cells (Fig 3A). In contrast, when probed with an antibody to the autophagosome marker MAP1LC3B (which reacts with two protein variants, LC3-I and -II), levels were higher in cells from the knockout animals than from the control mice, particularly in the case of the LC3-II species. This was particularly apparent in the CD4^+^ T and CD8^+^ T cell populations but rather less so in B cells. The LC3-II species is derived from LC3-I by lipidation during autophagy (for review see [23]) and becomes associated with autophagosome membranes. Levels of the LC3-II species correlate with the number of autophagosomes in a cell [24]; therefore, an increase in the levels of LC3-II in lymphocytes in the GIMAP6^fl/fl^CD2Cre mice is consistent with an increase in the number of autophagosomes in cells from these knockout animals. As these studies had been performed using lymphocyte lysates generated from pooled animals (routinely three in each group), CD4^+^ T, CD8^+^ T and B cell populations were also isolated from individual mice (one cell type per mouse) to allow statistical analysis of the LC3-II/ACTIN ratios. CD4^+^ T, CD8^+^ T and B cells from GIMAP6^fl/fl^CD2Cre knockout mice showed significantly higher LC3-II/ACTIN ratios than the corresponding cells from the control GIMAP6^fl/fl^ animals (Fig 3B).

An increase in the level of LC3-II is an indicator of an increase in the number of autophagosomes in cells but this could arise due to either an increased autophagic flux or a downstream block in the flux. Analysis of the levels of a second protein, SQSTM1, can help to distinguish between these possibilities. If autophagic flux is increased, levels of SQSTM1 will reduce, as it is transported through the autophagic pathway for eventual degradation. In contrast, if the autophagic pathway is inhibited, SQSTM1 levels will tend to increase. Therefore, lysates from CD4^+^ T, CD8^+^ T and B cells from GIMAP6^fl/fl^CD2Cre mice and GIMAP6^fl/fl^ animals were analysed for SQSTM1 levels by western blotting (Fig 3A). The knockout animals showed no reduction in the level of the protein compared with the control mice, suggesting that the absence of GIMAP6 did not increase flux of SQSTM1 through the autophagic pathway. However, although the total amount of SQSTM1 did not appear to be reduced in the knockout animals, it was now split into two distinct species with an additional species of slightly lower mobility being observed, in addition to the normal product (large arrow in Fig 3A). SQSTM1 has been reported to undergo phosphorylation at several sites in response to a range of signals. Of note, phosphorylation at residue serine-405 (corresponding to serine-403 in humans) has been demonstrated to occur during disruption of autophagic processes [25]. An antibody specific for this phosphorylated residue in SQSTM1 was therefore used to test the possibility that this was the modification associated with the *Gimap6* gene inactivation. As shown in Fig 3A, lysates from all three lymphocyte populations from the knockout animals contained a species reactive with this antibody and having a mobility consistent with it being the upper band observed using the general anti-SQSTM1 antibody discussed above. This species was undetectable in lysates from the control mice, indicating a specific accumulation of the phosphoserine-405 variant of SQSTM1 in the lymphocytes lacking GIMAP6. Interestingly, phosphatase treatment of cell lysates from either GIMAP6^fl/fl^ or GIMAP6^fl/fl^CD2Cre mice resulted in a band shift to lower molecular weight of the SQSTM1 species (S3 Fig), suggesting that even in control animals some residues in SQSTM1 are phosphorylated, although not at serine-405. Overall, it is apparent that GIMAP6 regulates autophagic turnover of SQSTM1 in a process involving phosphorylation of the protein. However, because only small changes in the levels of total SQSTM1 were observed, it remains unclear whether GIMAP6 ablation increases or reduces autophagy in these cells.

As a second method of investigating the effect of GIMAP6 on flux through the autophagic pathway, freshly isolated splenic CD4^+^ T cells from both GIMAP6^fl/fl^ and GIMAP6^fl/fl^CD2Cre animals were cultured in the absence or presence of the autophagy inhibitor BafA. BafA causes a block late in autophagy and so prevents LC3 turnover. Thus, the difference between the accumulation of LC3-II in the presence or absence of the inhibitor is a measure of the flux through the pathway. A typical result obtained is shown in Fig 3C. In the absence of BafA, consistent with the data in Fig 3A, LC3-II levels were higher in the cells from the knockout GIMAP6^fl/fl^CD2Cre animals than from the control animals. In the presence of the inhibitor, a large increase in LC3-II levels was observed in the cells from the control animals, consistent with the blockade of autophagic flux by the inhibitor. In contrast, treatment of cells from knockout animals with BafA caused only a small (statistically insignificant—Fig 3C right-hand panel) increase in LC3-II levels compared with untreated cells. Since the background levels of LC3 were so different between cells from control and knockout animals, it is difficult to draw firm conclusions about GIMAP6 function from these data. Nevertheless, if BafA inhibition can cause an increase, albeit small, in LC3-II levels in the knockout cells, this would indicate that the absence of GIMAP6 does not cause a complete block in the autophagic pathway.

### The impact of GIMAP6 ablation on mitochondrial load

**Fig 4:**
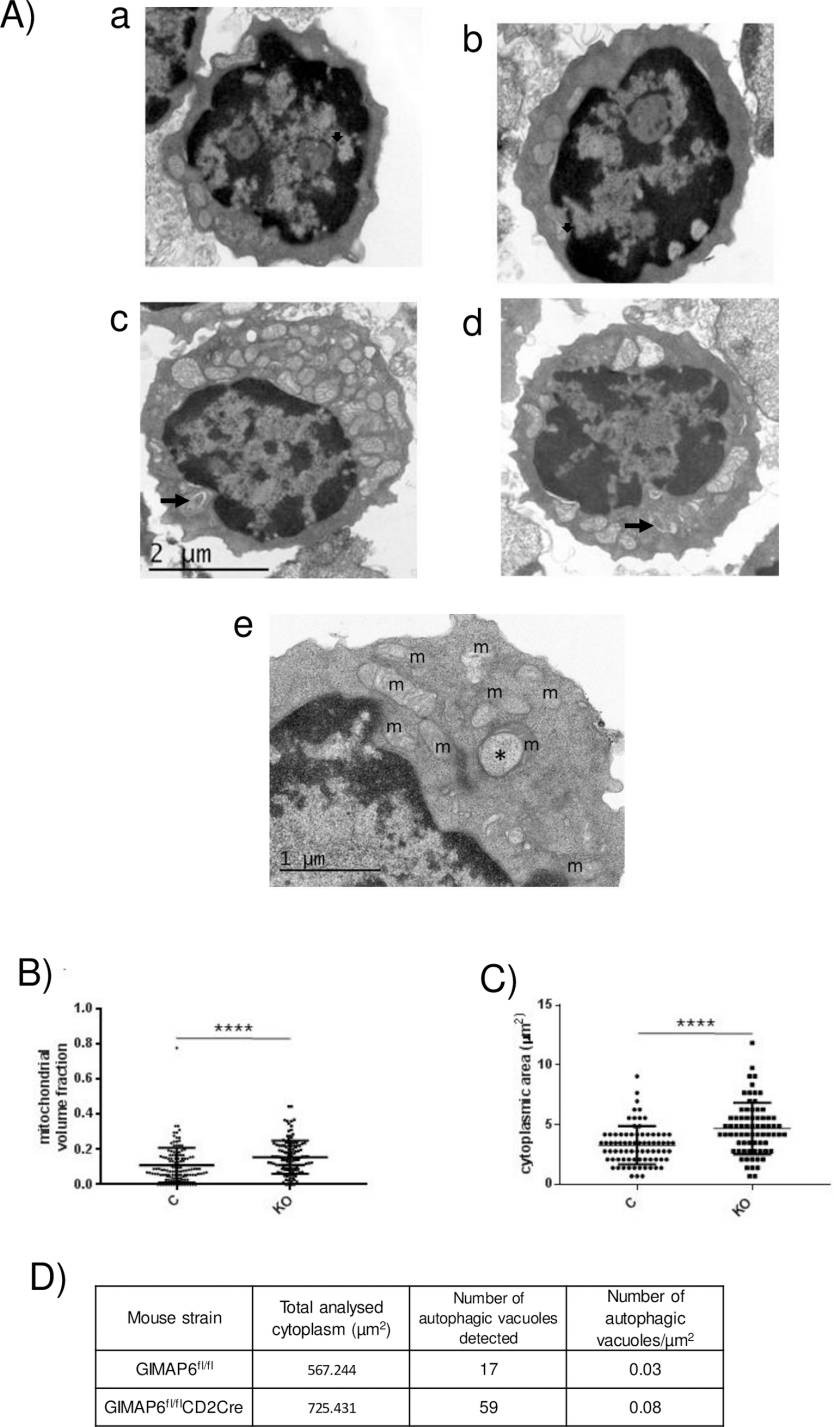
CD4^+^ T cells from GIMAP6^fl/fl^CD2Cre mice have increased cytoplasmic area, mitochondrial volume fraction and number of autophagic vacuoles. Electron micrographic images derived from CD4^+^ T cells were prepared and analysed as described in the Materials and Methods section. Panel A—Electron micrographs showing examples of CD4 cells purified from GIMAP6^fl/fl^ mice (micrographs a and b) and GIMAP6^fl/fl^CD2Cre mice (micrographs c and d). The arrows on micrographs c and d indicate autophagosomes. Micrograph e shows a higher power of a cell from a GIMAP6^fl/fl^CD2Cre animal to illustrate mitochondria (m) and a mitochondrial spheroid (*). Panel B—Calculated mitochondrial/cytoplasmic volume ratios (n = 160 cell profiles for cells from GIMAP6^fl/fl^ mice (C) and n = 158 cell profiles for GIMAP6^fl/fl^CD2Cre mice (KO) Panel C–Measured cytoplasmic area per nucleated cell profile (n = 78 nucleated cell profiles from GIMAP6^fl/fl^ mice (C) and n = 73 nucleated cell profiles for GIMAP6^fl/fl^CD2Cre mice (KO). For Panels B and C ****p<0.0001 (Mann Whitney U test). Panel D–Tabulation of the total analysed cytoplasmic area and the number of autophagic vacuoles observed from CD4^+^ T cells from GIMAP6^fl/fl^ mice (160 cell profiles) and GIMAP6^fl/fl^CD2Cre mice (158 cell profiles).

Autophagy serves not only to recycle intracellular components in cells in a relatively non-specific manner, but also has a key role in clearing specific organelles. For example, the autophagic pathway is required to remove mitochondria from reticulocytes during their maturation to erythrocytes [26], a process termed mitophagy. Similarly, a developmentally regulated reduction in mitochondrial content dependent upon mitophagy has been proposed for the T cell lineage at the stage where cells migrate from the thymus to secondary lymphoid tissues (blood, spleen, lymph nodes); in mice in which components of the autophagic system have been ablated, a reduction in T cell numbers is observed [27,28], perhaps because of the accumulation of excess reactive oxygen species. To address whether *Gimap6* gene disruption had an effect on mitochondria present in lymphocytes, transmission electron microscopy (EM) was used to measure the mitochondrial/cytoplasmic volume ratio of CD4^+^ T cells from control and knockout animals. Fig 4A shows low power EM fields of CD4^+^ T splenocytes isolated from GIMAP6^fl/fl^ (panels a and b) and from GIMAP6^fl/fl^CD2Cre mice (panels c and d). As summarised in Fig 4B, there was a highly significant increase in the mitochondrial/cytoplasmic volume ratio in CD4^+^ T cells from the GIMAP6^fl/fl^CD2Cre mice compared with the GIMAP6^fl/fl^ controls. In addition, occasional mitochondrial spheroids were observed in the GIMAP6^fl/fl^CD2Cre derived cells, which are suggestive of mitochondrial malfunctioning (Fig 4A panel e—asterisked) [29,30], although a single spheroid was also observed in the GIMAP6^fl/fl^ derived cells. The electron microscopic analyses also showed, firstly, an increase in the cytoplasmic area per nucleated cell (Fig 4C) and, secondly, an increase in the number of autophagosomes per cell profile (Fig 4D) (in agreement with the increased levels of LC3-II measured—Fig 3), although, owing to the low numbers seen, the latter could not be analysed statistically. For illustrative purposes, however, autophagosomes are arrowed in panels c and d of Fig 4A. The observed increase in cytoplasmic area (and in cell diameter)–see below) is consistent with previous work indicating that inhibition of autophagy can affect the regulation of cell size [31].

To directly compare mitochondrial load between GIMAP6^fl/fl^CD2Cre mice and GIMAP6^fl/fl^ mice, freshly isolated CD4^+^ T cells from mouse spleens of the two genotypes were incubated with Mitotracker® Green FM which selectively enters and is retained by mitochondria and flow cytometric analysis used to quantify the dye retained in the cells. CD4^+^ T cells from GIMAP6^fl/fl^CD2Cre animals retained significantly more Mitotracker than cells from control mice (Panel A in S4 Fig). Thus, in agreement with the EM data, CD4^+^ T cells from the GIMAP6^fl/fl^CD2Cre mice knockout have a greater mitochondrial load than the corresponding control mice. This data is consistent with an inhibition of autophagy (and thus probably also mitophagy) in the GIMAP6^fl/fl^CD2Cre mice. However, analysis of the mitochondrial/nuclear DNA ratio (Panel B in S4 Fig) failed to show any significant differences between the CD4^+^ T splenocytes from the two groups of animals, indicating that there was no change in the number of mitochondria between the cells. Moreover, analysis of the expression levels of two of the enzymes involved in mitochondrial fusion (MITOFUSIN-2) or fission (DYNAMIN RELATED PROTEIN 1—DRP1) did not show any difference between GIMAP6^fl/fl^ and GIMAP6^fl/fl^CD2Cre mice (Panel C in S4 Fig). However, it should be noted that both DRP1 and the mitofusins undergo regulatory post-translational modifications (see e.g. [32] for a review) that can have major effects on their activities without changes in the total levels of the proteins; If these modifications are changed in association with GIMAP6 ablation, it may provide an explanation for the observed increase in mitochondrial load in those cells.

### GIMAP6 is required for maintenance of normal peripheral T lymphocyte numbers

**Fig 5:**
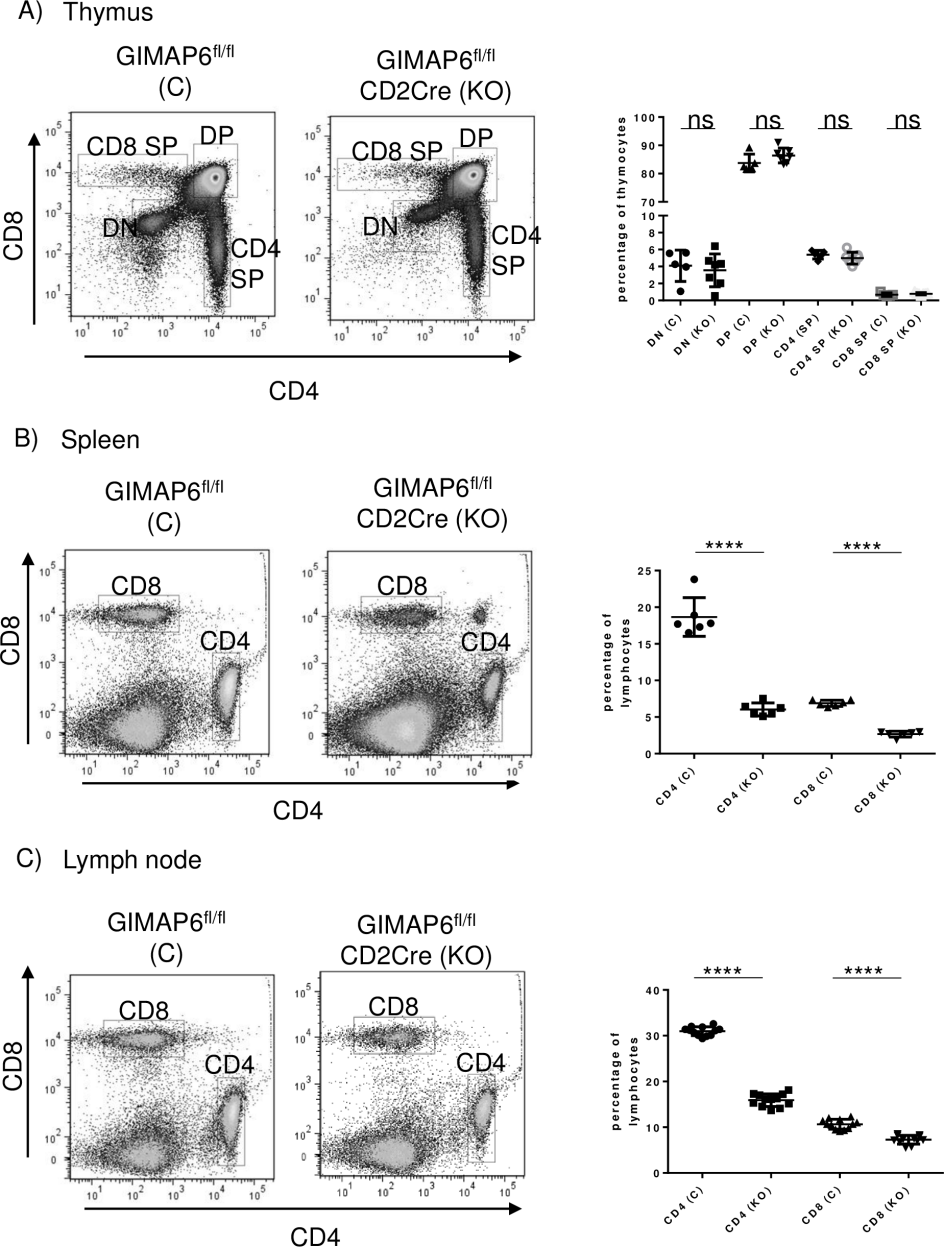
CD4 and CD8 cell analysis in GIMAP6^fl/fl^CD2Cre mice. Total cell suspensions were prepared from thymi, spleens and lymph nodes, were stained for CD4 and CD8 and analysed by flow cytometry. Nucleated cells were gated on the basis of FSC versus SSC, live versus dead and then for CD4 and CD8. Left-hand panels for each tissue show the gated cells. Nomenclature on the plots is: CD4 SP–single-positive CD4 cells; CD8 –single-positive CD8 cells; DP–cells positive for CD4 and CD8; DN–cells negative for both CD4 and CD8; CD4—CD4 cells; CD8 –CD8 cells. In each case, the left-most plot is from GIMAP6^fl/fl^ mice and the right hand plot from GIMAP6^fl/fl^CD2Cre mice. The graphs to the right show the calculated percentage of each cell population. GIMAP6^fl/fl^ mice are indicated by (C) and GIMAP6^fl/fl^CD2Cre by (KO). P values as follows: ns P>0.05; **** P<0.0001. n = 5–7 (unpaired 2-tailed Student’s t test).

As some GIMAP proteins have previously been shown to be important for the maintenance of normal lymphocyte numbers, the possibility that this might also be true for GIMAP6 was investigated. First, analysis of the percentages of thymus-resident CD4 and CD8 double-negative, double-positive and single-positive cells indicated no significant difference between GIMAP6^fl/fl^ and GIMAP6^fl/fl^CD2Cre animals (Fig 5A), a result similar to that previously seen for other GIMAPs [9,13,17,19]. In contrast to thymocytes, however, when lymphocytes from spleen and lymph nodes were examined, a highly significant reduction (to between 30 and 50% of control levels) in the percentages of CD4^+^ and CD8^+^ T cells in the tissues from the GIMAP6^fl/fl^CD2Cre mice was evident (Fig 5B and 5C). A similar reduction in CD4^+^ and CD8^+^ T cell numbers was observed in the two tissues (S5 Fig).

**Fig 6:**
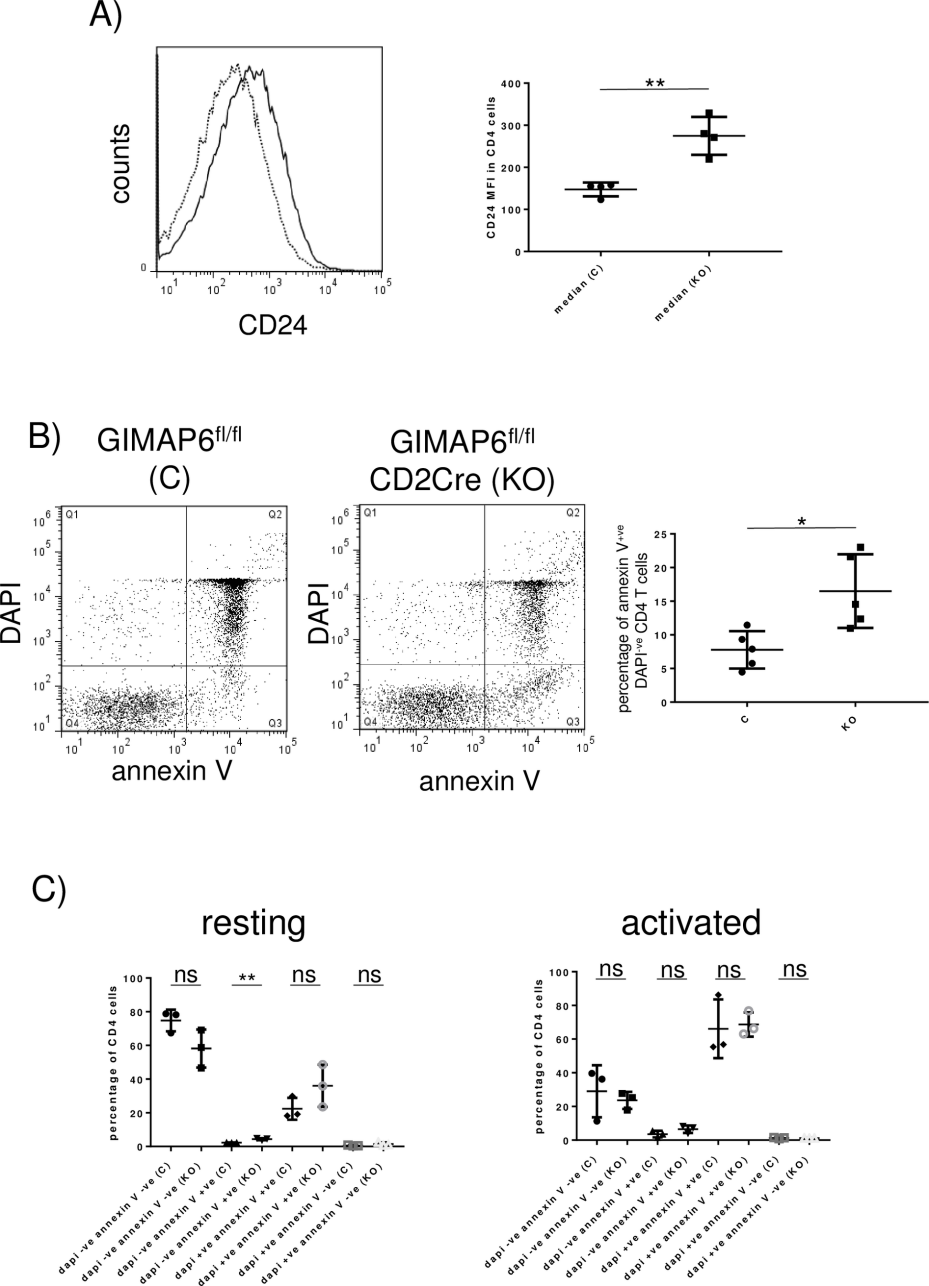
The reduced number of peripheral CD4^+^ T cells in GIMAP6^fl/fl^CD2Cre mice reflects an increase in apoptosis. A) Splenocytes isolated from GIMAP6^fl/fl^ and GIMAP6^fl/fl^CD2Cre animals were stained with fluorescent antibodies to CD4 and CD24. The left-hand panel shows the distribution of CD24 staining on CD4^+^ cells from single mice of the two genotypes (GIMAP6^fl/fl^–dotted line; GIMAP6^fl/fl^CD2Cre–solid line). The right-hand panel shows the median fluorescence intensity of CD24 staining of CD4^+^ cells. n = 4; **P<0.01. B) Freshly isolated splenocytes were stained with an APC-conjugated antibody to CD4 and PE- conjugated annexin V. The cells were then washed and stained with DAPI before flow analysis. The two dot-plots show typical staining patterns for a control (C) and knockout (KO) animal. The quadrants are as follows: Q1 –annexin V^-^ DAPI^+^; Q2 –annexin V^+^ DAPI^+^; Q3 –annexin V^+^ DAPI^-^; Q4 –annexin V^-^ DAPI^-^. The right-hand panel summarises the percentage of annexin V^+^ cells as a percentage of total DAPI^-^ CD4^+^ T cells for five animals in each group. ns P>0.05; ** P<0.01. C) CD4^+^ enriched naive splenocytes from GIMAP6^fl/fl^ and GIMAP6^fl/fl^CD2Cre mice were activated by maintenance on anti-CD3 antibody-coated plates in medium containing anti-CD28 and IL2 or were maintained in medium without activation as described in the Materials and Methods section. After 24 h cells were harvested and stained as in (B). The percentage of CD4^+^ T cells in each quadrant is indicated. n = 3 for each group; ns P>0.05; ** P<0.01.

There are two obvious possibilities as to the reason for the reduced number of peripheral T cells in the GIMAP6^fl/fl^CD2Cre mice: firstly, the output from the thymus is reduced, and secondly, the turn-over rate of circulating cells is increased, although the overall effect could be a combination of these. To try to separate the components two approaches were used. Firstly, we used the fact that early thymic emigrants express the surface antigen CD24 which reduces over time in circulating peripheral cells (see e.g.[33]). Splenocytes isolated from GIMAP6^fl/fl^CD2Cre and GIMAP6^fl/fl^ animals were stained with antibodies to CD4 and CD24 and the level of CD24 staining of CD4^+^ T cells assessed by flow cytometry. A typical staining distribution pattern is shown in the left hand panel of Fig 6A. The CD4 cells from the knockout mice showed higher levels of staining than those from the control animals. This result was borne out by analysis of multiple animals, where a consistent increase in the median fluorescence intensity was observed (Fig 6A right hand panel). These results would indicate that the CD4^+^ T cells from the knockout GIMAP6^fl/fl^CD2Cre animals had a younger age profile than those from the control animals, suggesting that thymic output was not reduced in the knockout animals but that the turnover rate of older cells was increased. As a second approach, the percentage of CD4^+^ T cells exhibiting signs of early apoptosis was measured in freshly isolated splenocytes by staining with annexin V and DAPI. In this assay, cells in early apoptosis appear as annexin V^+^ DAPI^-^. Typical staining profiles for GIMAP6^fl/fl^ and GIMAP6^fl/fl^CD2Cre animals are shown in the two left-most panels of Fig 6B. Analysis of multiple animals of each genotype (Fig 6B right-hand panel) indicated a statistically significant increase in the percentage of cells in the early apoptotic phase (annexin V^+^ DAPI^-^—corresponding to quadrant Q3) in knockout mice compared with the control animals, a result consistent with higher levels of apoptosis in the CD4^+^ T cells of the knockout animals, and thus with faster turn-over of the cells. A similar result was obtained when naïve CD4^+^ T cells were cultured in resting conditions for 24 h before analysis (Fig 6C left hand panel). Together these results suggest that the reduction of the numbers of circulating CD4^+^ T cells observed in the knockout animals arises because of increased rates of apoptosis rather than reduced production. Surprisingly, when naïve CD4^+^ T cells were activated *in vitro* by treatment with anti-CD3 and anti-CD28 for 24h before apoptotic analysis, cells from control and knockout animals behaved similarly, with a large increase in cell death (annexin V^+^ DAPI^+^) compared with resting cells (Fig 6C right hand panel) (a phenomenon known as activation-induced cell death–see e.g [34]).

### Effect of Gimap6 ablation on B lymphocytes

In published knockout models of both *Gimap1* [17] and *Gimap5* [13] reductions in the levels of B cells as well as T cells were observed. Therefore, B cell populations in the *Gimap6* knockout animals were also investigated. The changes which were observed (increases) are described in S6 and S7 Figs.

### Gimap6 ablation has only minor effects on T cell activation

**Fig 7:**
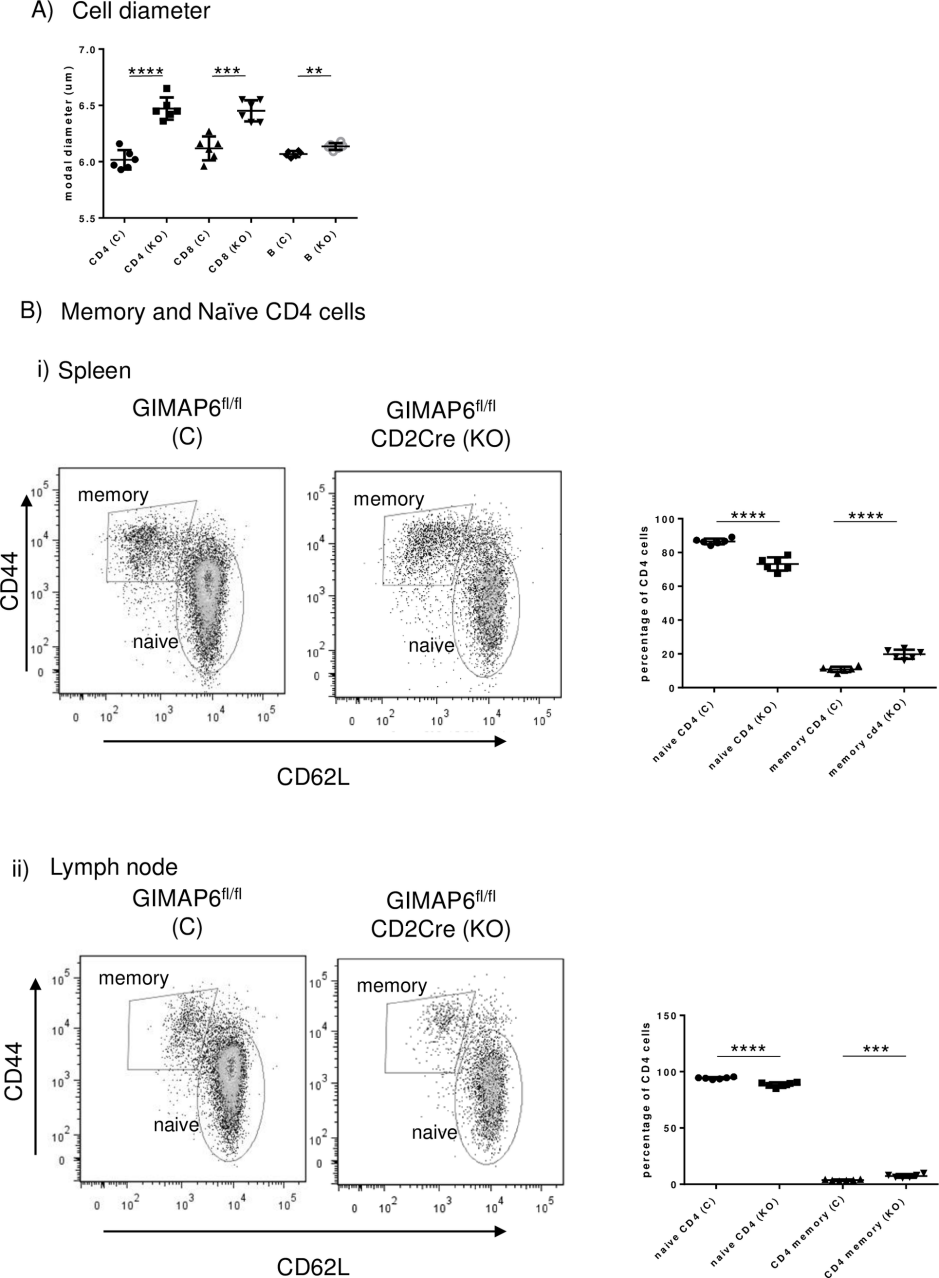
CD4^+^ cells from GIMAP6^fl/fl^CD2Cre mice show an increased size and a small increase in memory-like characteristics compared with GIMAP6 ^fl/fl^ mice. Panel A—The diameters of CD4^+^, CD8^+^ and B cell populations isolated from spleens of GIMAP6^fl/fl^ and GIMAP6^fl/fl^CD2Cre animals were measured using an electronic counter (CASY® Cell Counter + Analyser System—Schärfe System GMBH). Panel B–total splenic or lymph node-derived cells were stained for CD4 and then with antibodies to CD62L and CD44. Naïve cells were identified as CD62L^+^CD44^-^and memory cells as CD62L^-^CD44^+^. Graphs show each population expressed as a percentage of CD4^+^ cells. n = 6. P values as follows: **P<0.001 **** P<0.0001; n = 6 for each group (unpaired 2-tailed Student’s t test).

Previous work on *Gimap5* knockout mice and the spontaneous rat *lymphopenia* (*lyp*) mutation in *Gimap5* has indicated that the residual T lymphocyte populations are atypical compared to wild type [13,35]. Therefore, the CD4^+^ T and CD8^+^ T cells present in GIMAP6^fl/fl^CD2Cre mice were investigated in more detail. Firstly, as shown in Fig 7A, splenic CD4^+^ T, CD8^+^ T and, to a lesser extent, B cells purified from GIMAP6^fl/fl^CD2Cre animals had greater diameters than those from GIMAP6^fl/fl^ animals. Secondly, analysis of surface expression of CD62L and CD44 on splenic and lymph node CD4^+^ T cells showed a small but significant shift from CD62L^hi^CD44^lo^ surface expression on cells from GIMAP6^fl/fl^ mice towards a CD62L^lo^CD44^hi^ phenotype in cells from the GIMAP6^fl/fl^CD2Cre animals (Fig 7B). The changes in diameter and the surface marker expression suggest a modest shift away from the typical naïve phenotype. In addition, slightly more of the residual CD4^+^ T cells exhibit a more memory-like phenotype.

**Fig 8:**
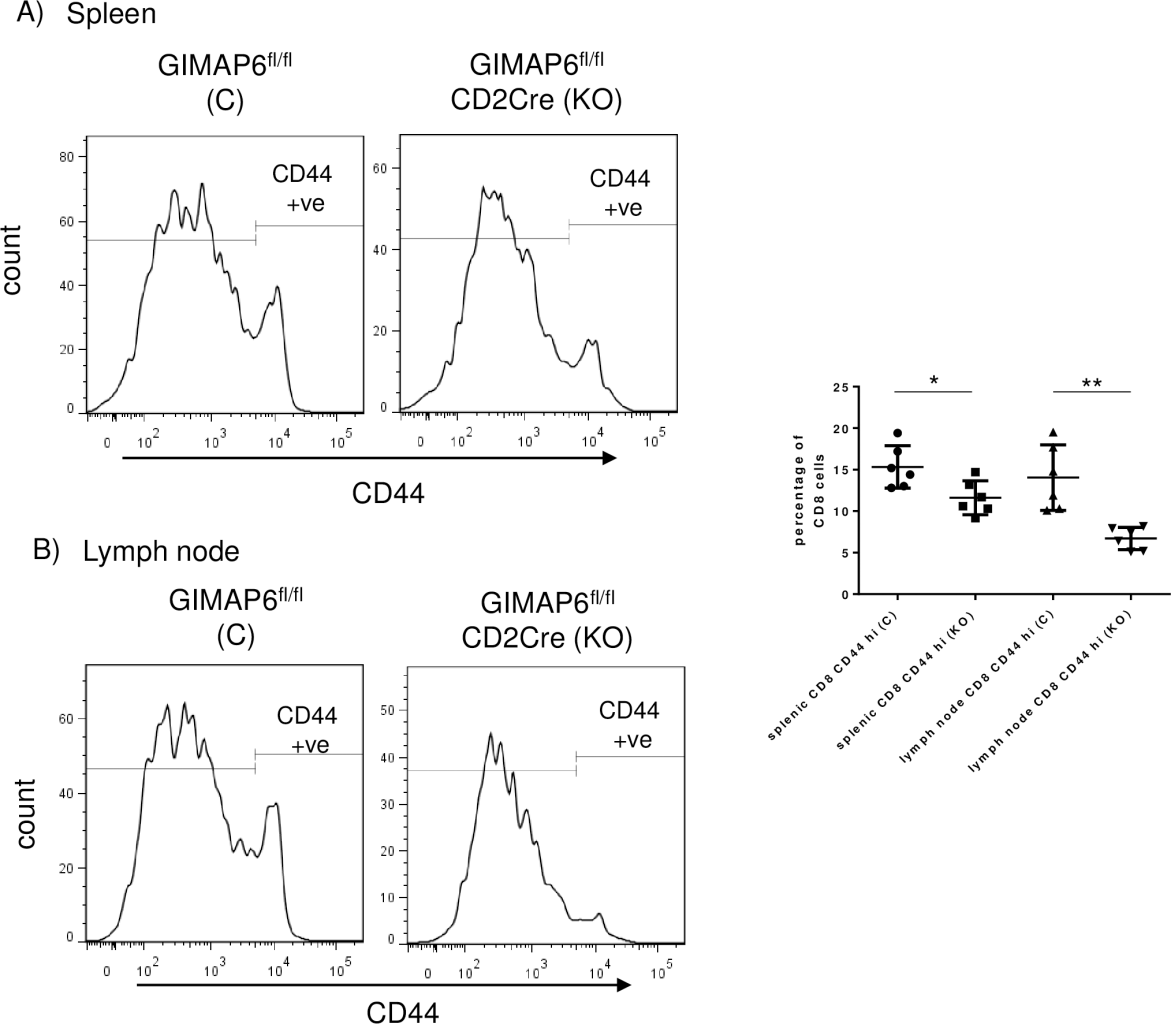
CD8^+^ cells from GIMAP6 ^fl/fl^ CD2Cre mice show a decrease in memory-like characteristics compared with GIMAP6 ^fl/fl^ mice. Total splenic or lymph node-derived cells were stained for CD8 and then with an antibody to CD44. The extent of staining of the CD8^+^ cells by CD44 for each group of cells is represented by histograms. For graphical analysis, CD44^+^ cells were gated as indicated from the histograms and plotted as a percentage of total CD8^+^ cells. n = 6. P values *p<0.05, **p<0.01.

In contrast to CD4^+^ T cells, CD8^+^ T cells showed only a very small CD44^hi^ CD62L^lo^ population in spleen and lymph node from either GIMAP6^fl/fl^ or Gimap6^fl/fl^CD2Cre mice. Therefore, to compare the CD8 populations of these animals, CD8^+^ T cells were analysed with respect to CD44 levels alone. In contrast to the situation with CD4^+^ T cells, a reduction in the percentage of CD44^hi^ cells in the CD8^+^ T cell population was observed in both the spleen and lymph nodes of the GIMAP6^fl/fl^CD2Cre mice compared with the GIMAP6^fl/fl^ animals (Fig 8). This was consistent with a reduction in either the memory or activated state of these cells.

To investigate further whether the T cells from the GIMAP6^fl/fl^CD2Cre mice showed any signs of activation, the surface expression of the early activation marker CD69 was measured on CD4^+^ T and CD8^+^ T cells. No significant difference in the levels of CD69 was observed between GIMAP6^fl/fl^ and GIMAP6^fl/fl^CD2Cre animals in either CD4^+^ T or CD8^+^ T splenocytes (Panels A and B in S8 Fig). In addition, no consistent change in the percentage of CD4^+^ T cells that were also CD25^+^ was observed between control and knockout mice, although this was rather variable between experiments. However, an increase in the percentage of FoxP3^+^ and CD25^+^FoxP3^+^ cells was consistently observed in the knockout animals compared with control animals (Panel C in S8 Fig), suggesting that these cells may be slightly more resistant to cell death than the other groups of CD4^+^ T cells.

**Fig 9:**
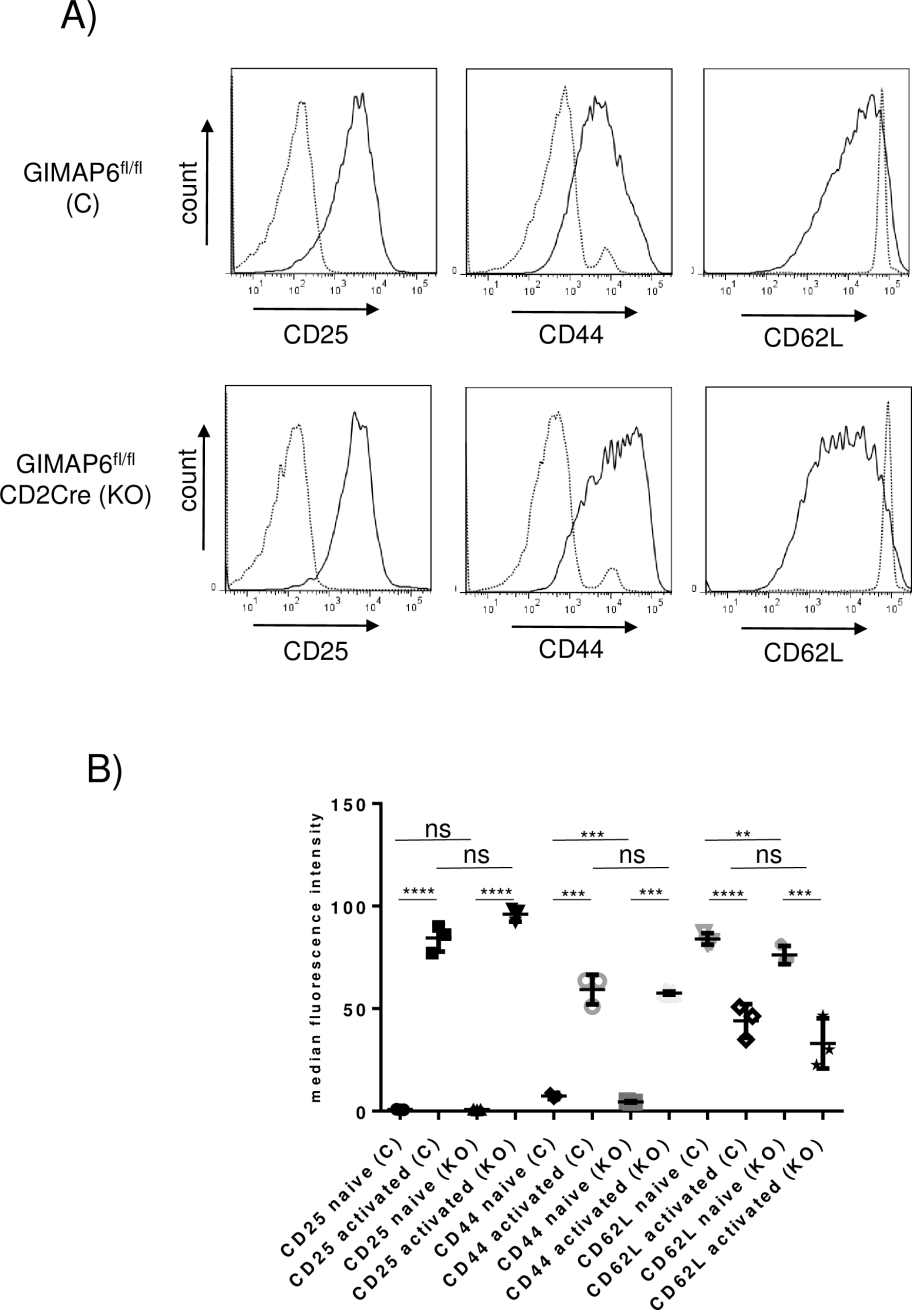
Activation of CD4^+^ splenocytes from GIMAP6^fl/fl^CD2Cre mice is comparable with those from GIMAP6^fl/fl^ mice. A) CD4^+^ enriched naïve splenocytes from GIMAP6^fl/fl^ and GIMAP6^fl/fl^CD2Cre mice were activated by maintenance on anti-CD3 antibody-coated plates in medium containing anti-CD28 and IL2 or were maintained in medium without activation. After 72h, cells were stained to measure expression of CD25, CD62L and CD44 to assess the degree of activation. On the histograms dotted lines indicate naïve cells and solid lines, activated cells. B) The median fluorescence intensities of the individual stains from three mice in each group is presented graphically. Unpaired 2-tailed Student’s t-test P values ns p>0.05 ***p<0.001; ****p<0.0001. The experiment was performed twice.

The ability of the CD4^+^ T cell population to respond to deliberate activation *in vitro* was also investigated. A typical set of results is shown in Fig 9. CD4^+^ T cells from either GIMAP6^fl/fl^ (control) or GIMAP6^fl/fl^CD2Cre (knockout) animals maintained *in vitro* for 72h in the absence of IL2 or anti-CD3 and anti-CD28 antibodies (i.e. non-activated) showed high levels of cell-surface CD62L and, respectively, low and undetectable levels of CD44 and CD25 (dotted lines in the panels in Fig 9A). In contrast, in *in vitro* anti-CD3/CD28-activated CD4^+^ T cells from both control and knockout mice, CD25 and CD44 cell surface expression was strongly induced, and levels of CD62L were reduced (solid lines in the panels in Fig 9), suggesting that there is no impairment of the ability of CD4^+^ T cells from the knockout animals to be activated. No statistical differences in the extent of CD25 or CD44 induction or CD62L reduction at the cell surface were observed between the cells from the control and the knockout animals (Fig 9B).

To summarise, it appears that the T cells from the GIMAP6^fl/fl^CD2Cre mice, though reduced in number, are relatively normal (but slightly enlarged), the majority being in a resting and non-activated state, although there is some suggestion of homeostatic expansion. Consistent with this conclusion, CD4^+^ T cells from these mice responded normally to activation *in vitro*; moreover, the mice themselves were able to generate essentially normal T cell-dependent antibody responses, although the primary response appeared somewhat laggardly (S9 Fig).

### Rapid appearance of phosphoserine-405 SQSTM1 but not of LC3-II accumulation after Gimap6 ablation

The data summarised in Figs 3 and 4, together with the observation that GIMAP6 is re-located to autophagosomes on starvation ([20] and Fig 1) provide strong support for the hypothesis that GIMAP6 is intimately involved in the process of autophagy. However, as the percentage and number of both splenic and lymph node T cells were reduced in the GIMAP6^fl/fl^CD2Cre mice, the possibility remained that at least some of the results obtained could be due to the selection of residual populations of lymphocytes with changed properties. We therefore concluded that it would be instructive/informative to study the impact of GIMAP6 loss in T cells shortly after ablation of the gene.

**Fig 10:**
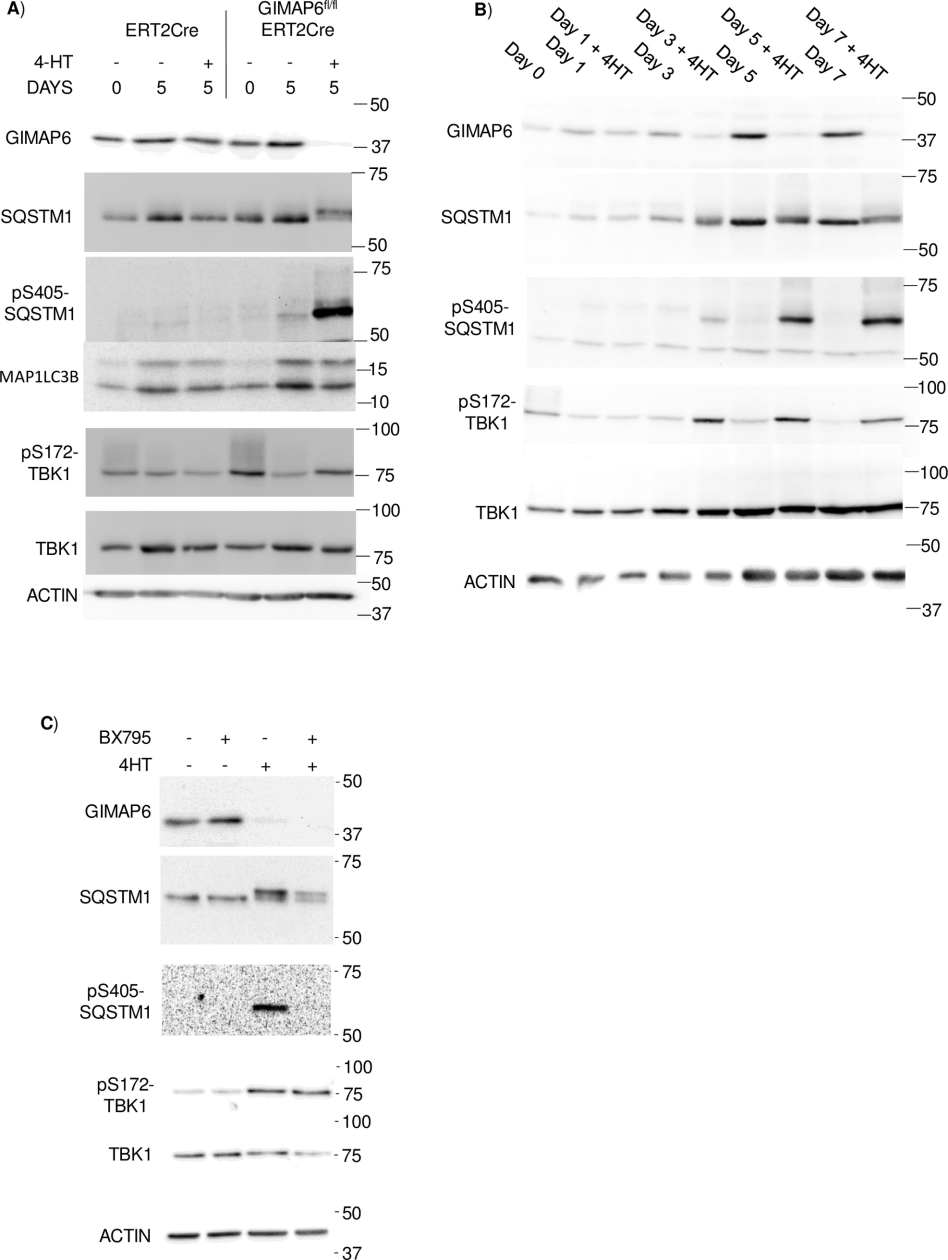
Effect on the expression of selected proteins of Gimap6 ablation in vitro from GIMAP6^fl/fl^ERT2Cre CD4^+^ T cells. Panel A) Enriched CD4^+^ cell fractions were prepared from spleens of ERT2Cre or GIMAP6^fl/fl^ERT2Cre mice. The cells were maintained in vitro for five days in the presence or absence of 200 nM 4HT. Cell lysates were prepared and analysed by SDS polyacrylamide gel electrophoresis with the indicated antibodies. Panel B) Enriched CD4^+^ cell fractions were prepared from spleens of GIMAP6^fl/fl^ERT2Cre mice. The cells were maintained in vitro in the presence or absence of 4HT for the indicated times before preparation of cell lysates and western analysis with the indicated antibodies. The electrophoretic mobilities of marker proteins are indicated. Panel C) Enriched CD4^+^ cell fractions were prepared and incubated with or without 200 nM 4HT for 4 days. TBK1 inhibitor BX795 (1 μM) was then added to the cells as indicated and incubation continued for 6h. Cell lysates were prepared and proteins analysed by western blotting. In all panels, the electrophoretic mobility of marker proteins is indicated. All results were confirmed in at least two experiments.

Our laboratory has previously reported the use of an inducible knockout system to ablate the *Gimap1* gene in a temporally controlled manner [36] using a tamoxifen-regulated Cre recombinase. A similar approach was adopted here to create a temporally controllable *Gimap6* knockout system. Mice carrying the ERT2Cre transgene were crossed to GIMAP6^fl/fl^ animals to generate GIMAP6^fl/fl^ mice carrying a single copy of the ERT2Cre gene (termed GIMAP6^fl/fl^ERT2Cre). Primary cells from these mice were used *in vitro* to study the acute effects of ablation of *Gimap6*. CD4^+^ T cells from the mice and from mice carrying a single copy of the ERT2Cre gene (as controls) were cultured with or without the active tamoxifen analogue 4-hydroxytamoxifen (4HT) for five days. This treatment had no effect on the levels of GIMAP6 protein in CD4^+^ T cells from the ERT2Cre mice. In contrast, in cells from the GIMAP6^fl/fl^ERT2Cre animals, immunoreactive GIMAP6 protein was reduced to barely detectable levels (Fig 10A), consistent with the targeted deletion of the *Gimap6* gene by the 4HT-activated ERT2Cre, and the subsequent turnover of already synthesised *Gimap6* mRNA and GIMAP6 protein. When some key autophagic markers were examined by western blotting, S405-phosphorylation of SQSTM1, and the associated additional SQSTM1 species previously observed in the GIMAP6 CD2Cre animals, were found to be present in the 4HT-treated GIMAP6^fl/fl^ERT2Cre-derived CD4^+^ cells but not in any other cell/treatment combination. However, in contrast to the situation in the GIMAP6^fl/fl^CD2Cre animals (described above), no increase in the levels of LC3 was observed. This difference may possibly be explained by the fact that the two gene ablation systems are assessing different timescales (see Discussion*)*.

### In GIMAP6-ablated CD4^+^ T cells, S405-phosphorylation of SQSTM1 is partially mediated by tank-binding kinase 1

Three enzymes have been suggested to be involved in S405-phosphorylation of SQSTM1: casein kinase II [25], ULK1 [37] and tank-binding kinase 1 (TBK1) [38]. Notably, it has been shown that TBK1 plays an important role in mitophagy [38–40]. Associated with the activation of TBK1 is its phosphorylation on serine-172. We therefore used this phosphorylation event to investigate whether TBK1 was activated after targeted deletion of the *Gimap6* gene in 4HT-treated GIMAP6^fl/fl^ERT2Cre CD4^+^ T cells. Phosphorylation of the serine-172 residue was higher in these cells after five days of treatment with 4HT than in untreated cells of the same genotype or in untreated or treated cells from ERT2Cre animals (Fig 10A), indicating that the TBK1 enzyme had been activated. Although this result was somewhat complicated by the relatively high basal level of TBK1 phosphorylation observed in cells immediately following isolation, it suggested that TBK1 may be responsible for at least some of the observed phosphorylation of SQSTM1 at serine-405.

To investigate this further, the time-courses of GIMAP6 disappearance and the associated phosphorylation of SQSTM1 and TBK1 were followed after treatment of GIMAP6^fl/fl^ERT2Cre splenic CD4^+^ T cells with 4HT. GIMAP6 was detectably reduced after three days of treatment when compared with untreated cells (Fig 10B). At this time-point, phosphorylation of both SQSTM1 and TBK1 first became detectable above the background levels present in untreated cells. As the level of GIMAP6 reduced further between three and five days of treatment, levels of phosphorylation of both SQSTM1 and TBK1 increased. Beyond five days no further consistent changes were observed. Thus, activation of TBK1 and phosphorylation of SQSTM1 correlated temporally with the disappearance of GIMAP6. Moreover, when 4HT-treated GIMAP6^fl/fl^ERT2Cre-derived CD4^+^ T cells were additionally treated with the TBK1 inhibitor BX795 [41] for 6h after 4 days of 4HT treatment, S405 phosphorylation of SQSTM1 was essentially eliminated compared with vehicle-treated cells (Fig 10C). This provides strong evidence that TBK1 is at least partially responsible for the observed increase in S405-SQSTM1 phosphorylation following GIMAP6 ablation, although the functional significance of this to the regulation of autophagy remains unclear. Surprisingly, using the anti-SQSTM1 antibody, an upper species of SQSTM1 was still observed after BX795 treatment, suggesting the generation of additional SQSTM1 phospho-forms, presumably either at very stable phosphorylation sites or catalysed by enzymes other than TBK1.

## Discussion

The GIMAP family of GTPases has been shown to be required for a normal mammalian peripheral lymphocyte system. Studies of rats carrying a natural mutation of the *Gimap5* gene [11,12,42,43] and mice carrying targeted or chemically-induced mutations of the orthologous gene [13,14] have shown that it is required for the establishment/maintenance of normal T and (to a lesser extent) B cell lymphocyte populations. Similarly, mice carrying a targeted mutation of the *Gimap1* gene in the lymphoid lineages lack T cells [17,44], although in this case there seems to be an equally strong effect on B cells [36]. A third membrane-anchored member of the family (GIMAP3) has also been demonstrated to be involved in T cell maintenance, although only when also linked to inactivation of GIMAP5 [18]. By contrast, knock-out (KO) studies in mice have indicated that neither GIMAP4 [19] nor GIMAP8 [9] plays a unique role in T cells *in vivo* i.e. one that cannot be compensated for by other proteins. However, both have been shown to change the rate of passage of T cells through apoptosis in response to apoptotic stimuli *in vitro* (GIMAP4 [19,45]; GIMAP8 [9]). Interestingly, the data published thus far have suggested that, whilst the three membrane-anchored GIMAP proteins (GIMAP1, GIMAP3 and GIMAP5) play unique roles in T cell maintenance, soluble proteins (GIMAP4 and GIMAP8) do not, perhaps indicating some particular roles for the membrane-anchored members of the family. However, the studies we report here indicate that this is too simplistic an interpretation as GIMAP6, a soluble protein, is clearly critical for the maintenance of T cells in the periphery, although its function seems to be dispensable for B cell maintenance.

What is the relationship of GIMAP proteins to cell survival? Early work indicated protein-protein interactions between some GIMAPs and members of the BCL-2 family [7], suggesting a function in the regulation of apoptosis. Indeed, GIMAP5 was identified in a screen for proteins able to protect against okadaic acid-induced apoptosis [46,47]. Moreover, as discussed above, deletion of either the *Gimap4* or *Gimap8* genes reduces the sensitivity of T cells to apoptosis–inducing agents *in vitro* [9,19,45]. In addition, work published whilst this manuscript was in preparation has suggested a role for GIMAP6 in the regulation of apoptosis [48]. However in contrast to GIMAPs 3, 4 and 5 reported on by Nitta and co-workers [7], Ho and colleagues were unable to demonstrate an interaction of GIMAP6 with any obvious apoptosis-associated protein, raising the possibility that GIMAP6 may be inducing apoptosis by a distinct route. This possibility has been strengthened by genetic and cellular studies of the lymphopenias and cell death associated with GIMAP5 and GIMAP1 deficiencies [14,36,44] which cast doubt on the involvement of the BCL2-related apoptotic machinery in the lymphopenias.

Further clues as to how GIMAP proteins may function has come from studies of their intracellular locations. Work from our laboratory has indicated that GIMAP1 is located principally in the Golgi apparatus, and GIMAP5 on lysosomes and multivesicular bodies (MVBs) [49]. In addition, GIMAP2 (a GIMAP family member found in humans but absent from rodents) is located on lipid droplets to which it recruits GIMAP7 [50]. These results may suggest a role for the GIMAP proteins in vesicular or lipid droplet trafficking or localised regulation of organelles. In the latter regard, it has recently been demonstrated that GIMAP5 regulates the levels of intra-luminal calcium in lysosomes in T cells [51], although it has also been shown that GIMAP5 can regulate mitochondrial integrity from a remote location [52]. Consistent with a possible role in calcium homeostasis, it has been demonstrated that T lymphocytes from a rat strain bearing the *lyp* mutant *Gimap5* gene show impaired calcium signalling [53]. Further work suggesting another potential mode of action of GIMAP5 has very recently been published. In this work, GIMAP5 has been shown to regulate the recruitment of glycogen synthase kinase-3β to vesicular structures (possibly MVBs) to inactivate the kinase. In the absence of GIMAP5, inactivation of the enzyme is impaired, leading to a failure in T cell proliferation [54].

Can we fit our new data with GIMAP6 into any of these models? Firstly, we have previously demonstrated that human GIMAP6 is recruited to autophagosomal vesicles upon the induction of autophagy [20], and in the current study we confirm that this also applies to mouse GIMAP6. It seems likely, therefore, that recruitment of GIMAP6 to autophagosomes is key to its function, as it is conserved even by the mouse protein, despite it lacking the principal domain required for GABARAPL2 interaction. Thus, GIMAP6 is another member of the GIMAP family that may fulfil its function by association with membranes.

What is GIMAP6 doing at the level of autophagosomes? The accumulation *in vivo* of LC3-II in lymphocytes from GIMAP6^fl/fl^CD2Cre mice is suggestive of a change in the process of basal autophagy. Similarly, the increased mitochondrial/cytoplasmic volume ratio we report might reflect a failure of the autophagic pathway to clear faulty mitochondria. It is of interest to address the fact that the accumulation of LC3-II seen *in vivo* in the GIMAP6^fl/fl^CD2Cre model was not observed *in vitro* in the inducible knockout GIMAP6^fl/fl^ERT2Cre system; by contrast, the increase in S405-phosphorylation of SQSTM1 was prominent and already detectable by day 3 after initiation of gene ablation. We ascribe this difference between the two systems to timescale: we suggest that the developmentally-regulated ablation of *Gimap6* in GIMAP6^fl/fl^CD2Cre lymphocytes leads to a gradual accumulation of LC3-II-bearing autophagosomal vesicles over a long period (weeks) *in vivo*, whereas the period of days to which our inducible *in vitro Gimap6* KO system is limited is insufficient to display such a change. On the other hand, the rapid change in the phosphorylation state of SQSTM1 suggests that this feature is highly sensitive to change in autophagosomal efficiency, perhaps through being especially attuned to any disruption of mitochondrial quality control.

As we have demonstrated that the SQSTM1 phosphorylation is at least in part mediated by TBK1, it is possible that GIMAP6 affects the phosphorylation and activation of that enzyme. It has previously been reported that activation of PINK1 and PARKIN by mitochondrial depolarization can lead to TBK1 phosphorylation. If GIMAP6 modulates this process, this could result in a change in the consequential TBK1-mediated phosphorylation of SQSTM1, with downstream effects on both the processes of autophagy and mitophagy.

Disruption of autophagy by targeted ablation of the autophagy protein Atg5 has been demonstrated to result in increased CD8^+^ T cell death and a failure of both CD4^+^ and CD8^+^ T cells to proliferate after T cell receptor stimulation [55]. In addition, Atg7 is required not only for peripheral T cell survival but also for the loss of mitochondria that occurs as T cells passage from the thymus to the periphery [27]. These authors suggest that it is this failure to lose mitochondria which results in the peripheral T cell death. We similarly speculate that the peripheral T cell lymphopenia seen in the *Gimap6* knock-out mice arises because of a reduction in autophagic flux leading to a failure of normal mitochondrial clearance.

Although our data point strongly to a role for GIMAP6 in the autophagic pathway, the level at which that occurs remains unclear. Formation of autophagosomes (as assessed by MAP1LC3B accumulation and electron microscopy) occurs, suggesting that the early stages of autophagic vesicle formation proceed normally and that GIMAP6 therefore modulates autophagy later in the pathway. Whereas many proteins have been characterised that are involved in autophagosome formation/sealing, fewer have been characterised that are important later in the pathway. One of the best studied is RAB7. RAB7 is required for the late maturation of autophagosomes and possibly their fusion with lysosomes [56]. T cell-specific ablation of RAB7 results in the accumulation of the lipidated form of MAP1LC3B, and inhibitor-mediated blockade of autophagy causes only a small further increase in this lipidated protein [57], in a manner similar to that reported in this paper for GIMAP6. This suggests that GIMAP6 and RAB7 may act at similar points in the autophagy pathway. Recent work has supported this idea by demonstrating the recruitment of GIMAP4, GIMAP5 and GIMAP6 to the parasitophorous vacuoles of *Toxoplasma gondii* infected cells to facilitate fusion of the vacuoles to lysosomes for parasite destruction [58]. However, a more detailed analysis of the later steps of autophagy in lymphocytes lacking GIMAP6 will be required to identify the exact point at which the protein acts.

One of the key remaining questions is why lymphocytes, and in particular T cells, should have acquired an additional control mechanism for autophagy (i.e. GIMAP6) operating in a cell type-selective manner, given that autophagy is a universal process throughout eukaryotes. We are unaware of many precedents for this. It has been suggested that the microtubule-associated protein, MAP1B, may have a particular role in autophagy in neurons where it is expressed at high levels, although it is expressed widely in other cell types [59]. Similarly, pleckstrin homology domain containing family member 1, PLEKHM1, which has recently been shown to regulate autophagosome-lysosome fusion, although expressed widely, appears to be particularly important in the regulation of the function of osteoclasts [60]. It is possible that tailored additional control systems exist to meet the needs of particular cell types. In this regard, it is noteworthy that T and B cells undergo transitions between actively proliferating states and survival for long periods in a quiescent state, which causes large associated metabolic changes. Similarly, the cells also have to deal with huge variations in oxygen concentrations between blood, spleen and lymph nodes and within secondary lymph nodes themselves and need tight control of mitochondrial load/activity. As the process of autophagy/mitophagy is a key regulator of catabolism and mitochondrial function, higher-level controls of the system may have evolved under the pressure of such huge changes in demand. We would speculate that GIMAP6 is a key component of this higher-level control.

## Materials and methods

### Mice

Mice were bred and maintained in the Babraham Institute Biological Support Unit under Specific Opportunistic Pathogen Free (SOPF) conditions. After weaning, mice were transferred to individually ventilated cages with 1–5 mice per cage. Mice were fed CRM (P) VP diet (Special Diet Services) *ad libitum* and received poppy seeds, sunflower seeds or millet on cage-cleaning as part of their environmental enrichment. All mouse experimentation was approved by the Babraham Institute Animal Welfare and Ethical Review Body. All animal husbandry and experimentation complied with existing European Union and United Kingdom Home Office legislation and local standards. All mice were used experimentally between 8–12 weeks of age and were age- and sex-matched within experiments, although no sex-associated differences were observed in the results obtained.

### Generation of GIMAP6-deficient mice

A *Gimap6* targeting construct in which exons 2 and 3 were flanked by loxP sites (see Fig 2) was generated by recombineering [61], starting from clone RPC-97A20 from the C57BL/6 RPCI-23 BAC library. The construct also included a neomycin resistance cassette to allow positive selection in embryonic stem (ES) cells, and thymidine kinase and diphtheria toxin A cassettes to allow negative selection if required (summarised in Fig 2A). The construct was electroporated into C57BL/6J mouse-derived ES cells, transfectants were selected in G418-containing medium, and correctly targeted clones identified by PCR and Southern blotting. Positive clones were subsequently used for the establishment of mice carrying a correctly targeted *Gimap6* gene (termed the GIMAP6^fl/fl^ mouse line), during which process the neomycin resistance cassette was excised by passage through the C57BL/6 Flp-deleter mouse line to promote frt-frt recombination. Mouse establishment was performed by genOway (Genoway, Inc., Lyon, France). These mice were then crossed either to mice carrying the hCD2-iCre transgene [22] to promote *Gimap6* gene inactivation in the lymphoid system or to CreER^T2^ mice [62] to allow temporal deletion of the gene in isolated cells *in vitro*, using 4-hydroxytamoxifen (4HT).

### Measurement of T-cell dependent B cell responses in GIMAP6 knockout mice

GIMAP6^fl/fl^CD2Cre mice or control GIMAP6^fl/fl^ mice were immunised with 4-hydroxy-3-nitrophenylacetyl (NP)19–keyhole limpet hemocyanin (KLH; Biosearch Technologies, Novato, CA) adsorbed to alum as described previously [36]. Mice were bled at the indicated times, before boosting with soluble antigen and antibody titres determined by ELISA essentially as previously described [63] except that NP_23_-BSA was replaced by NP_20_-BSA.

### Establishment of stable HEK293 cells expressing mouse GIMAP6

HEK293 cells were maintained in DMEM/10% (v/v) fetal calf serum/100 units/ml penicillin/100μg/ml streptomycin. The mouse GIMAP6-encoding cDNA was cloned downstream of a myc tag-encoding sequence in the plasmid pCANmyc1. Mouse GIMAP6-expressing HEK293 clones were then established essentially as described previously [20], except that X-tremeGENE^™^ 9 DNA Transfection Reagent (Roche) was used instead of lipofectamine.

### Immunocytochemical localisation of GIMAP6 in stably transfected HEK293 cells

HEK293 cells expressing mouse GIMAP6 were plated on to poly-lysine coated coverslips in 6-well plates at 10^5^ cells/well. After 2 days, cells were either left untreated or were incubated for 90 minutes in starvation medium (140mM NaCl, 1mM CaCl_2_, 1mM MgCl_2_, 5.5mM glucose, 20mM HEPES pH 7.4, 1% (w/v) BSA) in the presence of 100nM bafilomycin A1 at 37°C. Cells were then washed with ice-cold phosphate-buffered saline (PBS), fixed by treatment with ice-cold methanol and stored at -20°C for 15 minutes. They were then washed with PBS, and blocked for 1h in PBS containing 5% (w/v) bovine serum albumin and 0.05% Triton-X100. The fixed cells were then incubated in a mixture of four in-house rat anti-mouse GIMAP6 antibodies (MAC431, MAC432, MAC434 and MAC436) and rabbit polyclonal antibodies to either MAP1LC3B or SQSTM1. Slides were washed in PBS/0.05% Triton-X100 and then treated with AlexaFluor 488-conjugated goat anti-rat IgG and AlexaFluor 568-conjugated goat anti-rabbit IgG. After extensive washing, the stained cells were mounted in Vectashield mounting medium with DAPI (Vector Laboratories) and viewed using a Zeiss Axio Imager.D2 system.

### Flow analysis of cell populations

Mice were killed by carbon dioxide asphyxiation and cervical dislocation. Tissues were excised into FACS buffer (PBS containing 0.5% (v/v) fetal calf serum), and dispersed by passage through 40 μm filters and red cells lysed using ACK buffer (Gibco). Cells were subsequently stained using combinations of fluorochrome-conjugated anti-mouse antibodies: CD4 PE-Cy5.5, CD8 PE-Cy7, CD44 FITC, CD4-APC, CD25-PE, CD25 APC-eFluor 780, and annexin V-PE (all purchased from eBioscience) and CD62L PE and Alexafluor-488 FOXP3 (Biolegend). Dead cells were identified using DAPI. Flow analysis was performed on the cells using either an LSR-II or a Fortessa-5 analyser (BD- Biosciences). Data analysis was performed using Flowjo version 7 (Flowjo, LLC).

### Enrichment for primary lymphocyte cell populations

Populations enriched for CD4^+^ T, CD8^+^ T or B splenic lymphocytes were routinely prepared by negative cell enrichment using Magnisort cell selection technology (Thermo Fisher Scientific). In the specific case of the purification of CD8^+^ T cells from individual mice, the Magnisort Mouse CD8 T cell enrichment kit was used. Enrichments for all cell types were routinely greater than 90%, except for CD4 (80–85%) and CD8 (70–80%) from GIMAP6CD2Cre mice, presumably reflecting the lower percentage of those cells in the starting populations from those animals.

### Culturing of primary naïve CD4^+^ T cells

Primary naïve splenic CD4^+^ T cells were isolated by use of a Magnisort mouse CD4 T cell enrichment kit (eBioscience), supplemented with anti-CD25-biotin. Isolated cells were maintained in RPMI/10% (v/v) fetal calf serum/50 μM 2-mercaptoethanol/5 μg/ml IL-7/100 units/ml penicillin/100 μg/ml streptomycin at 37°C in 5% (v/v) carbon dioxide. For *in vitro* inactivation of the *Gimap6* gene, cells were maintained in 200nM 4HT. For *in vitro* activation experiments, the naïve cells were plated in RPMI/10% (v/v) fetal calf serum/50 μM 2-mercaptoethanol/10 ng/ml IL-2/100ng/ml anti-CD28 (eBioscience) on to dishes pre-coated with anti-CD3 (eBioscience) for 24-72h as indicated. Resting cells were maintained in RPMI/10% (v/v) fetal calf serum/50 μM 2-mercaptoethanol/10 ng/ml IL-7. Cells were treated with either 1 μM BX795 (InvivoGen) or 100 nM Bafilomycin A (Sigma) as required.

### Analysis of proteins produced by enriched cell populations

SDS polyacrylamide gel electrophoresis and western blotting of total cellular extracts were performed as described previously [20]. The following primary antibodies were used: rat monoclonal anti-mouse GIMAP6 (generated in-house—either MAC 436 alone or a mixture of four different monoclonal antibodies MAC431, MAC432, MAC434, MAC436); mouse monoclonal anti-β-actin (Sigma); rat monoclonal anti-phosphoSQSTM1 (Ser403) (MBL); mouse monoclonal anti-SQSTM1 (Abnova); rabbit polyclonal anti-MAP1LC3B; rabbit monoclonal anti-phosphoTBK1 (Ser172); rabbit monoclonal anti-TBK1 (all from Cell Signalling Technologies). Rat monoclonal antibodies to other mouse GIMAP proteins were also generated in-house (GIMAP1 –MAC420; GIMAP4 –MAC415; GIMAP5 –MAC421; GIMAP7 –MAC448; GIMAP8 –MAC443; GIMAP9 –MAC433). Horseradish peroxidase-conjugated anti-mouse, anti-rat and anti-rabbit IgGs were purchased from Cell Signalling Technologies. Western blots were routinely developed using Immobilon Western reagents (Millipore Inc.), except for blots of actin when Supersignal West Pico Chemiluminescent Reagent (Thermo Scientific) was used. Blots were visualised using a G-box system (Syngene). Where required, band intensities were quantified using GeneTools software (Syngene).

### Electron microscopic examination of CD4^+^ T cells

CD4^+^ T-enriched cells were prepared by positive selection from spleen using Mouse CD4 (L3T4) MicroBeads and columns from Miltenyi Biotec. Resuspended cells were diluted with an equal volume of 4% (v/v) glutaraldehyde in 0.4M HEPES buffer pH7.4 at 4°C, recovered by centrifugation (15000g, 5 minutes) and then incubated at 4°C for 2h. The resulting cell pellet was then stored in 0.2M HEPES buffer at 4°C for some days. For electron microscopy, the cells were post-fixed in 1% osmium tetroxide, block stained in 1% uranyl acetate, dehydrated in ethanol, and embedded in epoxy resin. Thin sections were cut using a diamond knife and stained with uranyl acetate and lead citrate, and examined using a Jeol JEM 1400 transmission electron microscope. Images were taken using systematic random selection of fields, at 2500X primary magnification. The images were zoomed on screen using Photoshop, and point counting was used to estimate the cytoplasmic area per cell profile and the volume fraction of mitochondria in cytoplasm.

### Labelling of CD4^+^ splenic T cells with Mitotracker® Green FM

Freshly-isolated CD4^+^ T cell enriched fractions were incubated in 25 nM Mitotracker® Green FM in RPMI/10% (v/v) fetal calf serum//100 units/ml penicillin/100 μg/ml streptomycin at 37°C in 5% (v/v) carbon dioxide for 15 minutes. Anti-mouse CD4-APC was included in the incubation to simultaneously stain the CD4^+^ T cells. Uptake of the Mitotracker dye was quenched by addition of ice-cold PBS/0.5% (v/v) fetal calf serum. Cells were recovered by centrifugation and resuspended in the same buffer in the presence of DAPI (live/dead cell stain). Mitotracker fluorescence was then measured in CD4^+^DAPI^-^ T cells on a BD LSRII Flow Cytometer.

### Determination of mitochondrial DNA copies per nuclear genome

Total DNA was prepared from CD4^+^ T splenocytes from GIMAP6^fl/fl^ and GIMAP6^fl/fl^CD2Cre mice using a QIAamp DNA blood mini kit (Qiagen) as described by the manufacturer. qPCR was then performed on the purified DNA using a SYBR Green PCR master mix (Applied Biosystems) in a Biorad CFX-96 real time system. The primer pairs used were as described [64] comprising CTAGAAACCCCGAAACCAAA plus CCAGCTATCACCAAGCTCGT for the mitochondrial amplicon and ATGGGAAGCCGAACATACTG plus CAGTCTCAGTGGGGGTGAAT for the nuclear amplicon. The mitochondrial DNA to nuclear DNA ratio was then determined from the relative C_q_ values.

### Data analysis

Graphical analysis was performed using the software package GraphPad Prism (Graphpad Software Inc.). Unless indicated otherwise, sample groups were compared using the unpaired 2-tailed Student’s t-test.

## Supporting information

S1 Fig GIMAP6 localisation on cell starvation or treatment with BafA. (TIF)

S2 Fig GIMAP6 does not co-localise with the lysosomal marker LAMP1. (TIF)

S3 Fig SQSTM1 is phosphorylated in GIMAP6^fl/fl^ cells. (TIF)

S4 Fig Characterisation of the mitochondrial content of GIMAP6^fl/fl^CD2Cre mice. (TIF)

S5 Fig Splenic and lymph node lymphocyte numbers. (TIF)

S6 Fig Characterisation of B cell populations in GIMAP6^fl/fl^CD2Cre mice. (TIF)

S7 Fig Numbers of individual B cell populations in spleen and lymph node. (TIF)

S8 Fig Comparison of the levels of activation marker expression on CD4^+^ and CD8^+^ cells from GIMAP6^fl/fl^CD2Cre and GIMAP6^fl/fl^ mice. (TIF)

S9 Fig T-cell dependent generation of antibody responses in GIMAP6^fl/fl^CD2Cre mice. (TIF)
