# Supplementary figures and images for: GIMAP6 is required for T cell maintenance and efficient autophagy in mice
Source: PLoS One. 2018 May 2;13(5):e0196504. doi: 10.1371/journal.pone.0196504 (PMC5931655; doi:10.1371/journal.pone.0196504)

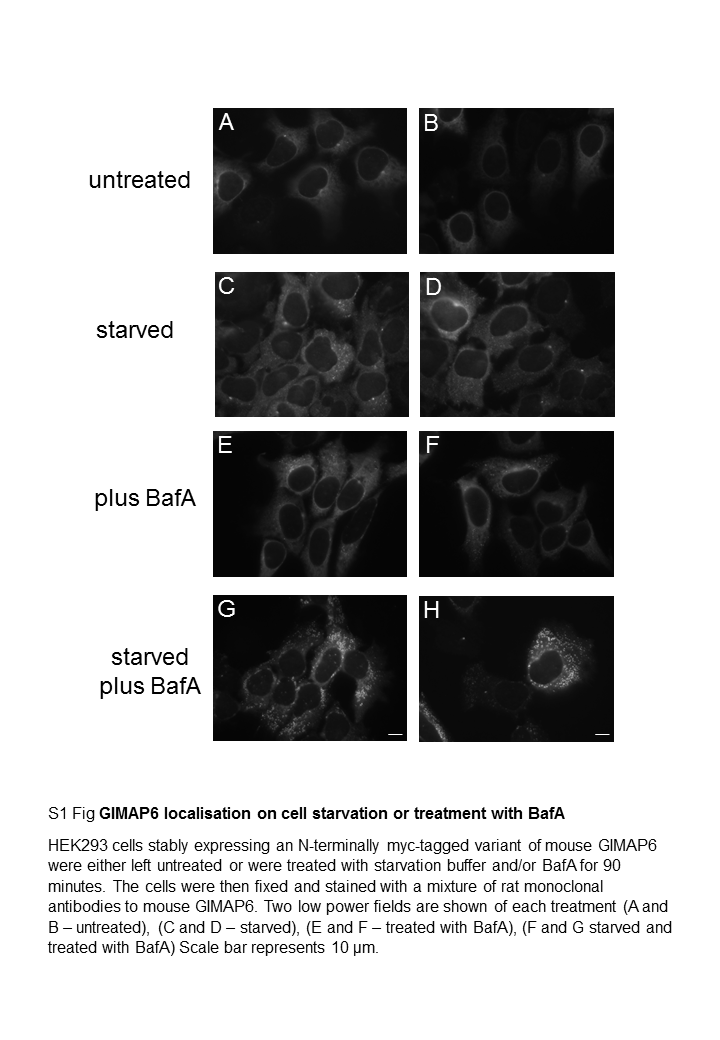

Supplement: S1 Fig — (TIF) [file pone.0196504.s001.TIF]

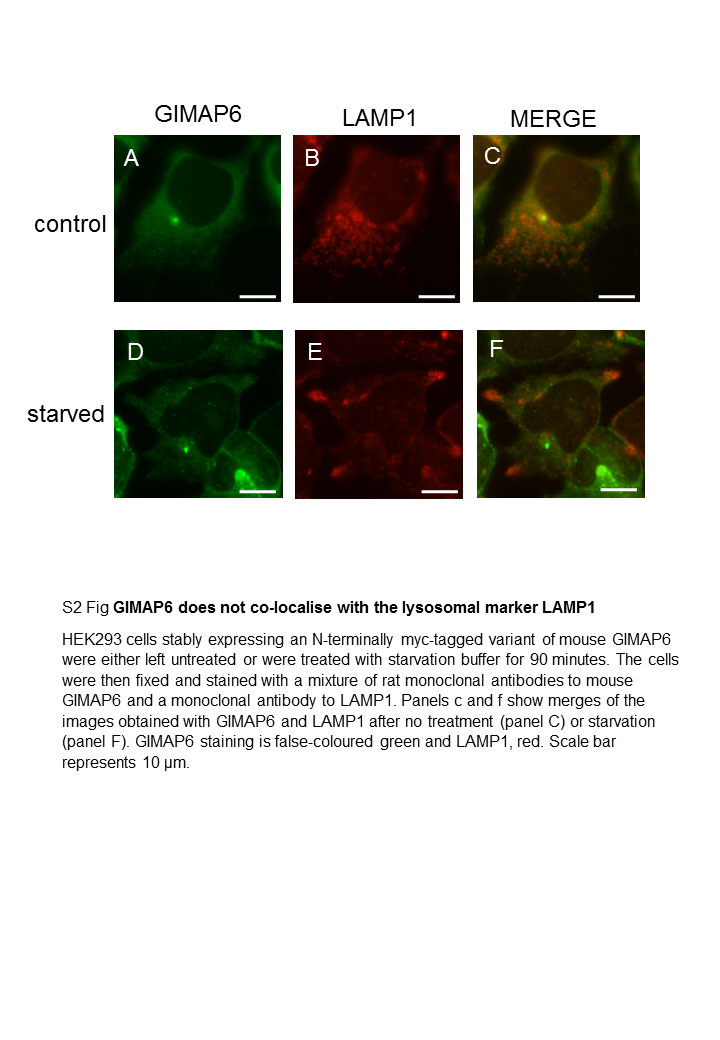

Supplement: S2 Fig — (TIF) [file pone.0196504.s002.TIF]

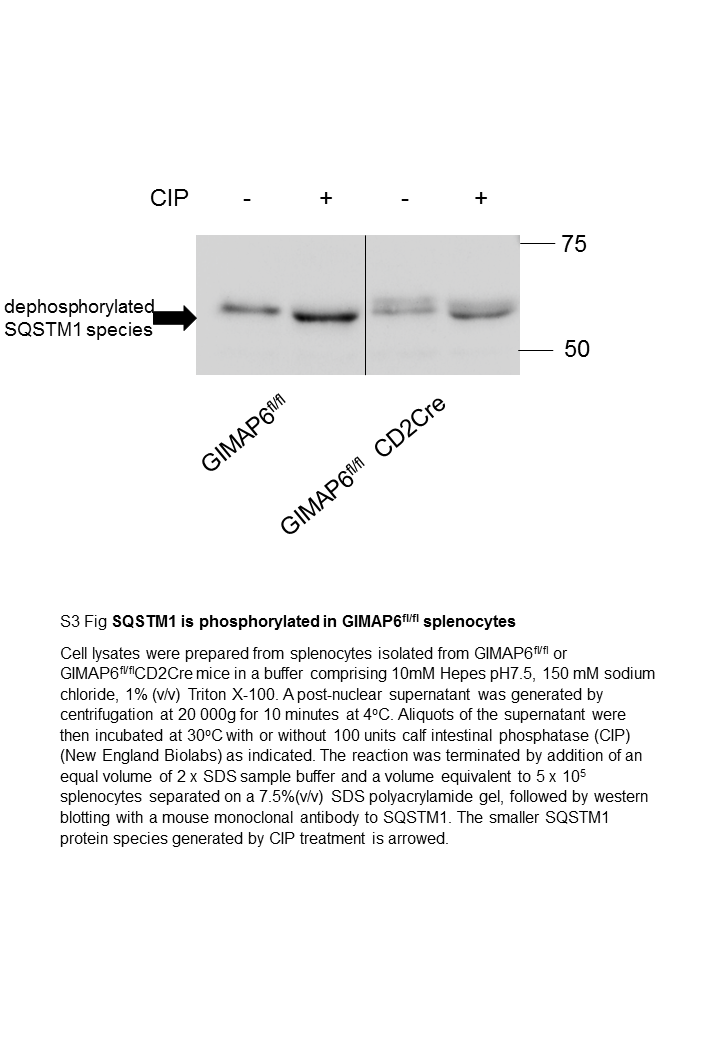

Supplement: S3 Fig — (TIF) [file pone.0196504.s003.TIF]

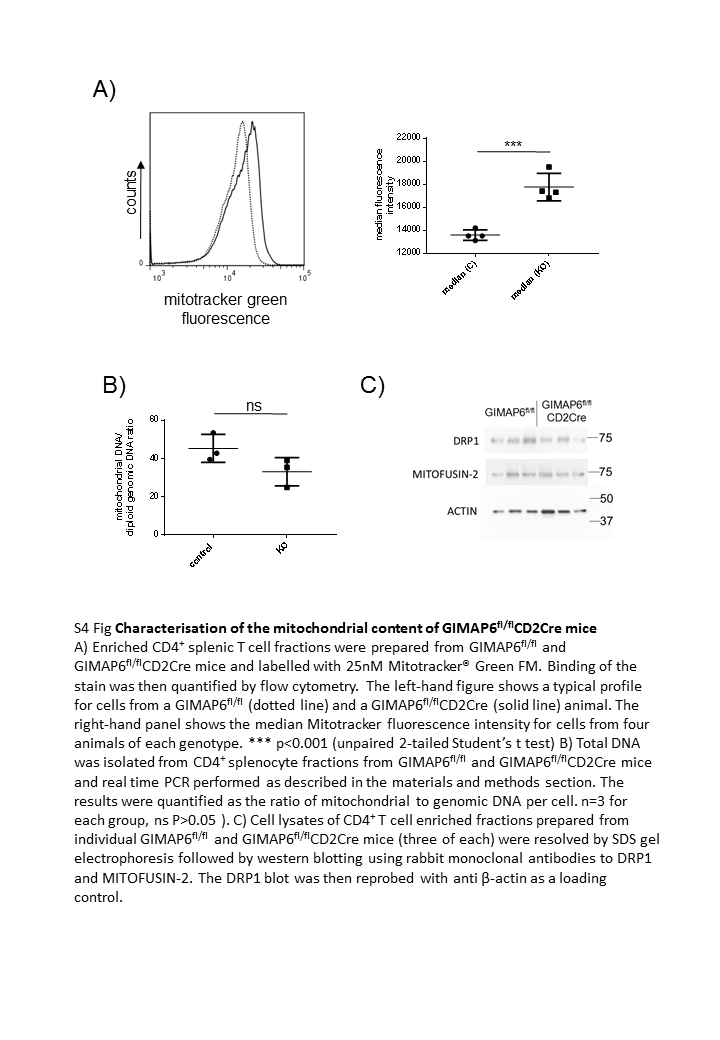

Supplement: S4 Fig — (TIF) [file pone.0196504.s004.TIF]

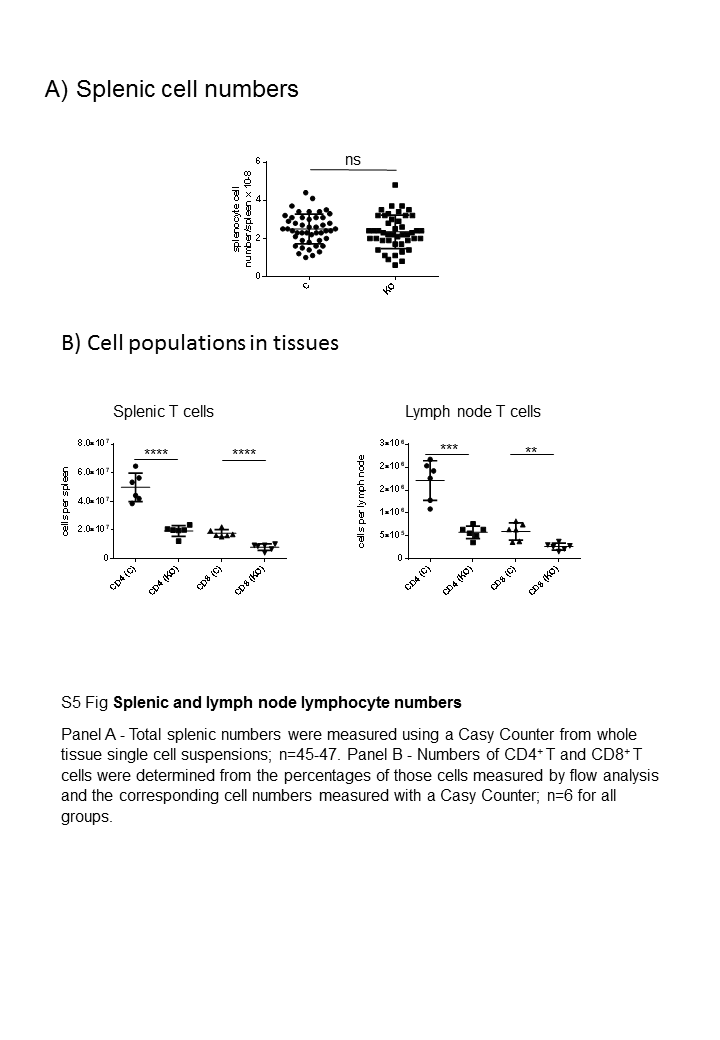

Supplement: S5 Fig — (TIF) [file pone.0196504.s005.TIF]

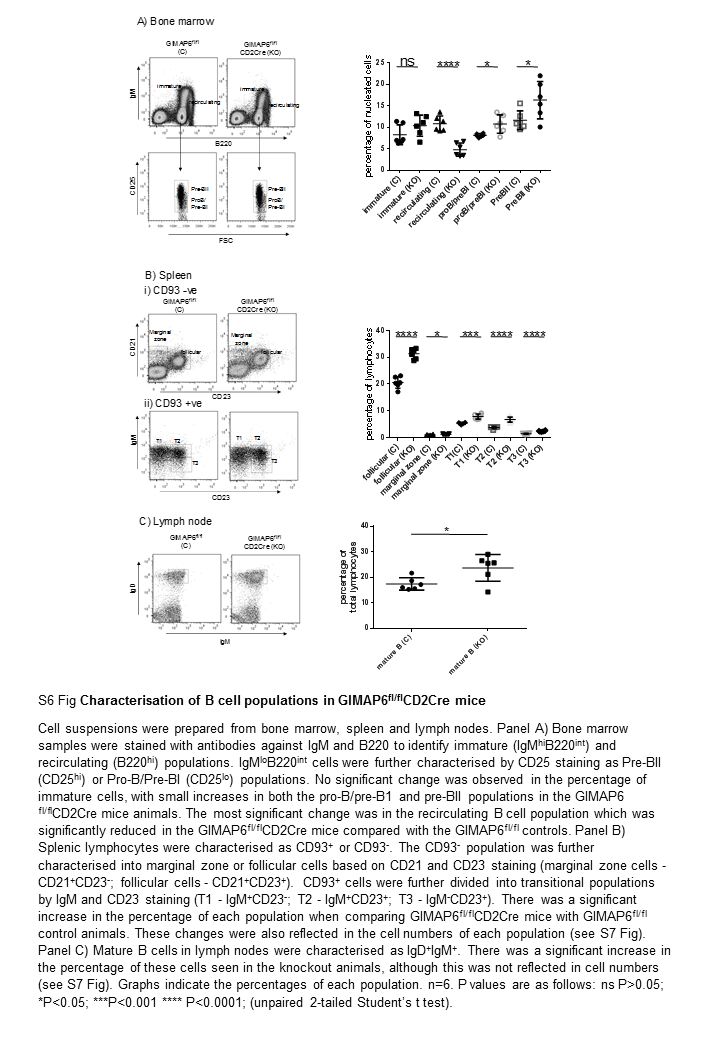

Supplement: S6 Fig — (TIF) [file pone.0196504.s006.TIF]

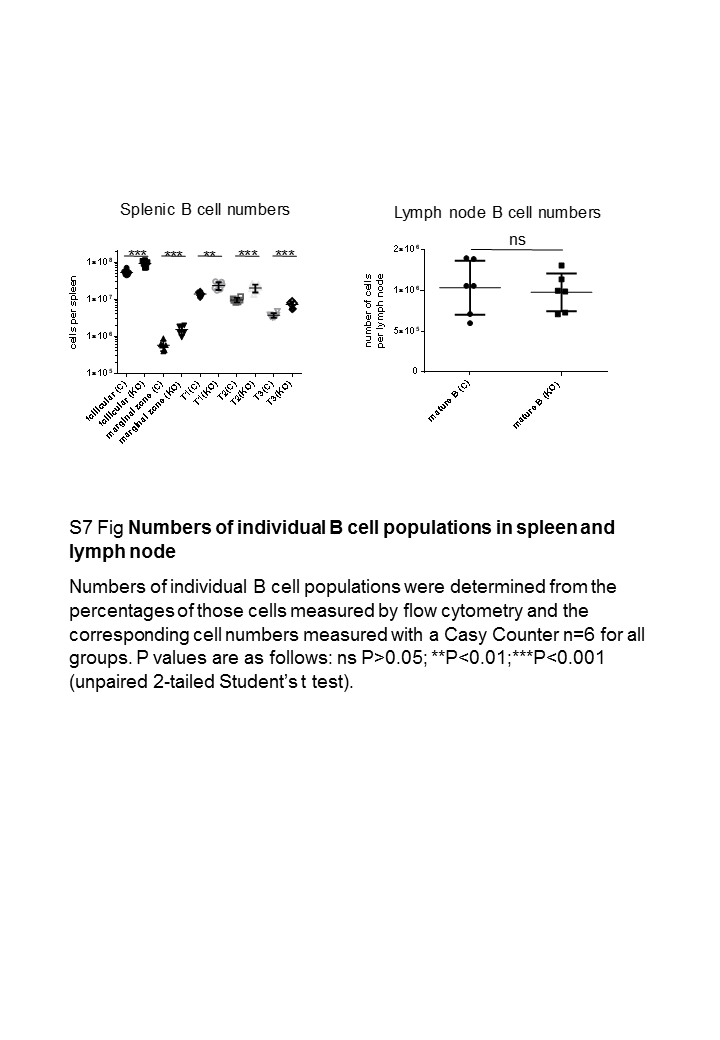

Supplement: S7 Fig — (TIF) [file pone.0196504.s007.TIF]

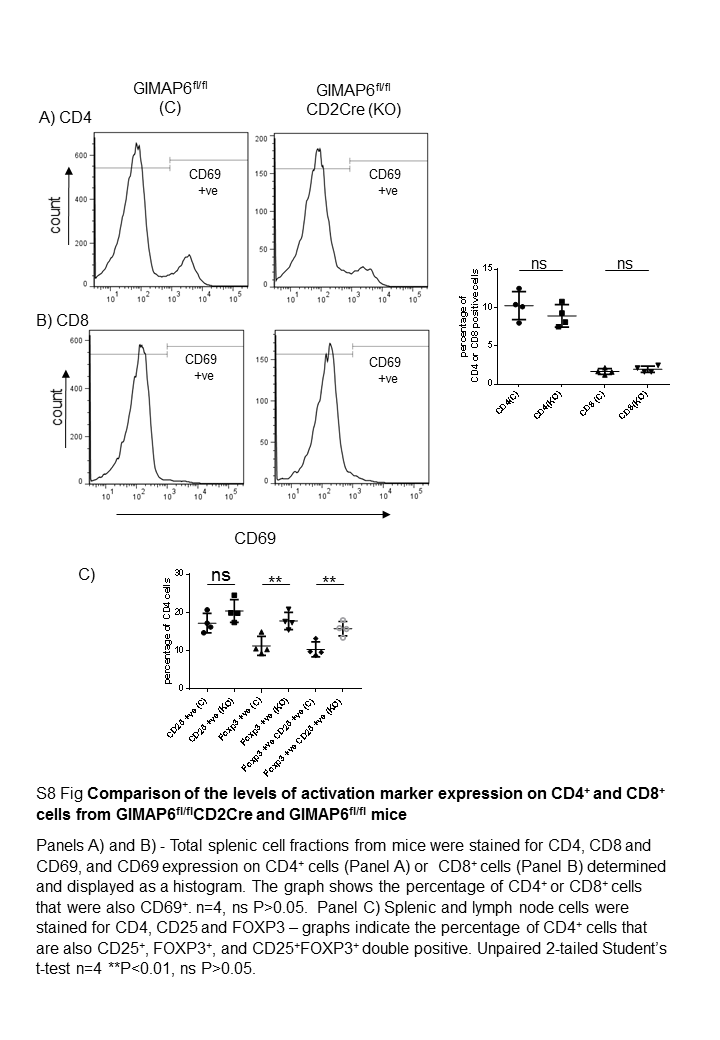

Supplement: S8 Fig — (TIF) [file pone.0196504.s008.TIF]

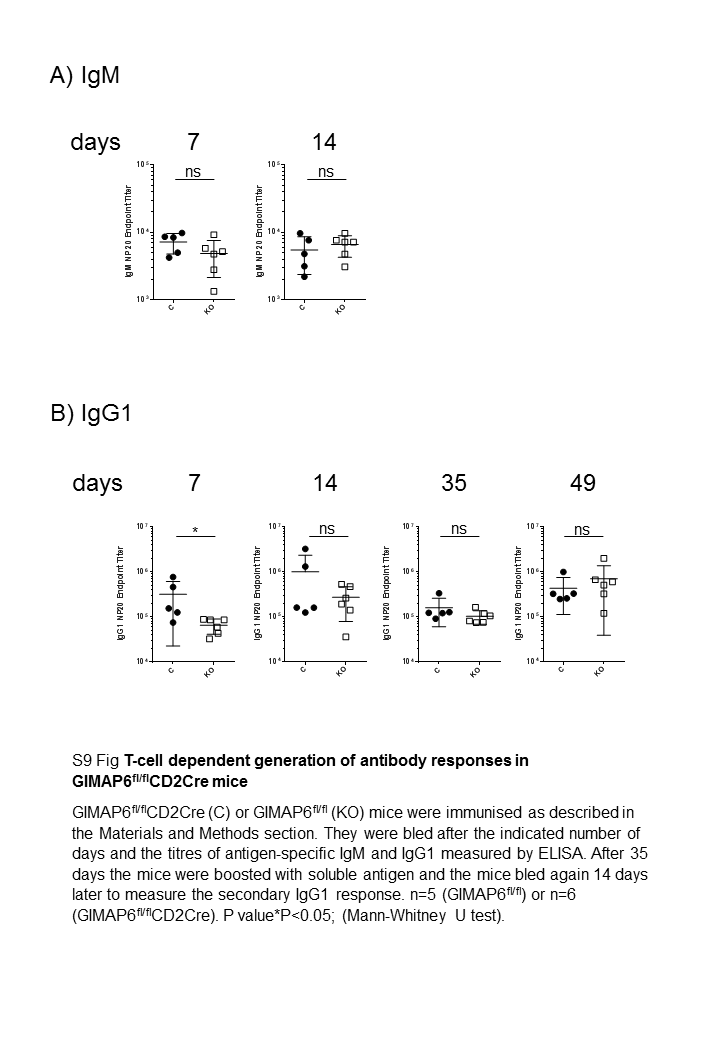

Supplement: S9 Fig — (TIF) [file pone.0196504.s009.TIF]
